# Electronically integrated microcatheters based on self-assembling polymer films

**DOI:** 10.1126/sciadv.abl5408

**Published:** 2021-12-17

**Authors:** Boris Rivkin, Christian Becker, Balram Singh, Azaam Aziz, Farzin Akbar, Aleksandr Egunov, Dmitriy D. Karnaushenko, Ronald Naumann, Rudolf Schäfer, Mariana Medina-Sánchez, Daniil Karnaushenko, Oliver G. Schmidt

**Affiliations:** 1 Institute for Integrative Nanosciences, Institute for Solid State and Materials Research Dresden (Leibniz IFW Dresden), 01069 Dresden, Germany.; 2 Max Planck Institute of Molecular Cell Biology and Genetics, Transgenic Core Facility, Pfotenhauerstrasse 108, 01307 Dresden, Germany.; 3 Institute for Metallic Materials, Institute for Solid State and Materials Research Dresden (Leibniz IFW Dresden), 01069 Dresden, Germany.; 4 Material Systems for Nanoelectronics, Chemnitz University of Technology, 09107 Chemnitz, Germany.; 5 Research Center for Materials, Architectures and Integration of Nanomembranes (MAIN), Rosenbergstraße 6, TU Chemnitz, 09126 Chemnitz, Germany.; 6 Nanophysics, Faculty of Physics, TU Dresden, 01062 Dresden, Germany.

## Abstract

Existing electronically integrated catheters rely on the manual assembly of separate components to integrate sensing and actuation capabilities. This strongly impedes their miniaturization and further integration. Here, we report an electronically integrated self-assembled microcatheter. Electronic components for sensing and actuation are embedded into the catheter wall through the self-assembly of photolithographically processed polymer thin films. With a diameter of only about 0.1 mm, the catheter integrates actuated digits for manipulation and a magnetic sensor for navigation and is capable of targeted delivery of liquids. Fundamental functionalities are demonstrated and evaluated with artificial model environments and ex vivo tissue. Using the integrated magnetic sensor, we develop a strategy for the magnetic tracking of medical tools that facilitates basic navigation with a high resolution below 0.1 mm. These highly flexible and microsized integrated catheters might expand the boundary of minimally invasive surgery and lead to new biomedical applications.

## Teaser

Self assembled catheters with deep sub-mm diameters deliver liquids, carry out micromanipulation, and sense magnetic fields.

## INTRODUCTION

Minimally invasive surgery is an integral part of modern health care (*1*) in which catheters occupy a key position. Cardiovascular and, later, cerebrovascular catheters have been steadily improved since their initial demonstration in the early 20th century (*2*) and, today, enable interventions including the removal of blood clots, the delivery of implants such as stents (*3*), or the targeted administration of drugs (*4*). Applying catheter-based interventions, patients are subjected to lesser direct trauma due to smaller surgical entry points. These interventions reduce the risk of medical complications, and the recovery time is greatly minimized, although requiring well trained and experienced medical practitioners. Benefiting from the development in the material sciences, modern catheters have a wide variety of shapes and sizes (*5*) and are routinely used in clinics worldwide. These devices are optimized to achieve ideal mechanical performance while being maneuvered within a network of vesicles by a surgeon. Nowadays, the frontier of surgical tool development is advanced by devices with integrated electronic and robotic capabilities. Taking advantage of modern semiconductor processing technologies and novel materials, integrated instruments have recently enabled in situ sensory diagnostics (*6*, *7*). In particular, vascular catheters are subject to diverse development efforts. The classical challenge of catheter steering is, today, addressed not only by pull-wire systems but also with soft-actuator solutions based on conductive polymer (*8*) and hydraulic actuators (*9*) as well as magnetic actuation. The latter is widely explored throughout academia (*10*, *11*) and the clinical sector, where it is used in robotic systems for cardiac ablation (*12*). Other commercial robotic systems, including the Da Vinci Robotic System (*13*) or Monarch Platform for robotic catheter control (*14*) provide medical practitioners with superhuman precision and control. These systems can further integrate sensors for noninvasive navigation (*15*, *16*) and have been established as powerful instruments within their respective fields of application. More recently, integrated catheters based on flexible and stretchable electronics have emerged to offer versatile, soft, and lightweight devices with high integration density over multiple size scales. Upcoming tools are expected to offer functionalities such as real-time physiological (*17*–*19*) and biochemical sensing (*20*, *21*), position tracking (*22*), and local tissue treatment or stimulation (*23*). The common strategy in achieving these active medical tools up to now was to equip classical commercial catheters or similar tubular structures with separately fabricated electronic components, predominantly flexible electronics (*20*, *24*, *25*). This approach, however, does not allow a substantial miniaturization of catheters while preserving a high integration density of complex electronics and wide range of functionalities. This shortcoming is confirmed by the lack of reported devices with a characteristic size smaller than 0.7 mm, hinting at the high manufacturing effort needed for the realization of small-scale, functional electronic catheter systems.

**Fig. 1.**
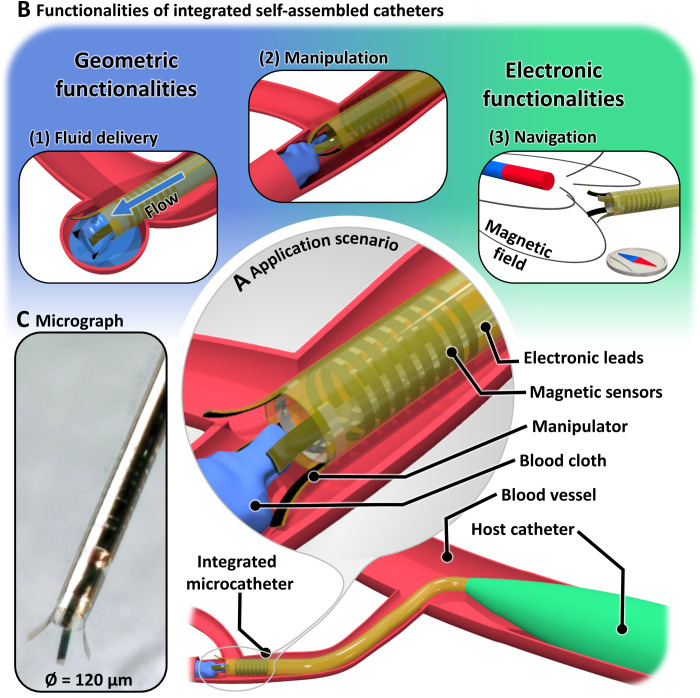
Concept of the ISAC. (**A**) The ISAC is equipped with opposing actuated digits and a magnetic sensor. Both are controlled through integrated electronic leads. The ISAC is deployed into a microscopic blood vessel from a regular microcatheter. The host accommodates electronic and fluidic connections. (**B**) Three potential application scenarios that highlight the functionalities of ISACs: (1) fluid embolization of an aneurysm, (2) retrieval of a blood clot with the integrated manipulator, and (3) navigation using the integrated magnetic sensor as electronic compass. (**C**) Micrograph showing the tip of an Ø = 120-μm ISAC. Photo credit: Boris Rivkin, Institute for Integrative Nanoscience, Leibniz IFW Dresden.

**Fig. 2.**
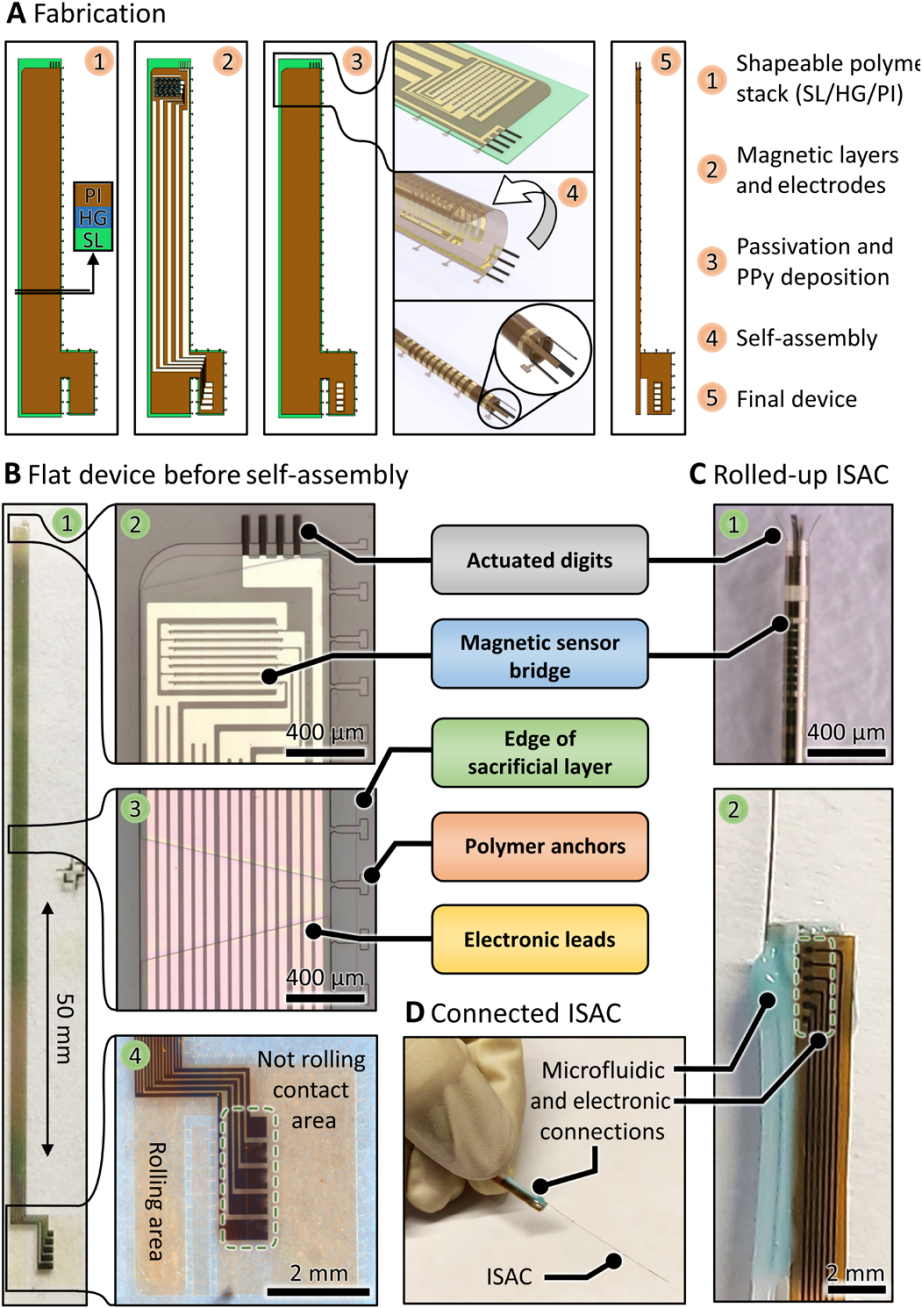
Fabrication routine and features of ISACs. (**A**) Fabrication of ISACs as stacks of planar thin films and subsequent self-assembly into rolled-up tubular devices. (**B**) Flat device before assembly. (**C**) Rolled-up ISAC with integrated electronic components (1) and electronic and fluidic connections (2). (**D**) The final devices with electronic and fluid connections fully released from the handling substrate. Photo credit: Boris Rivkin, Institute for Integrative Nanoscience, Leibniz IFW Dresden.

**Fig. 3.**
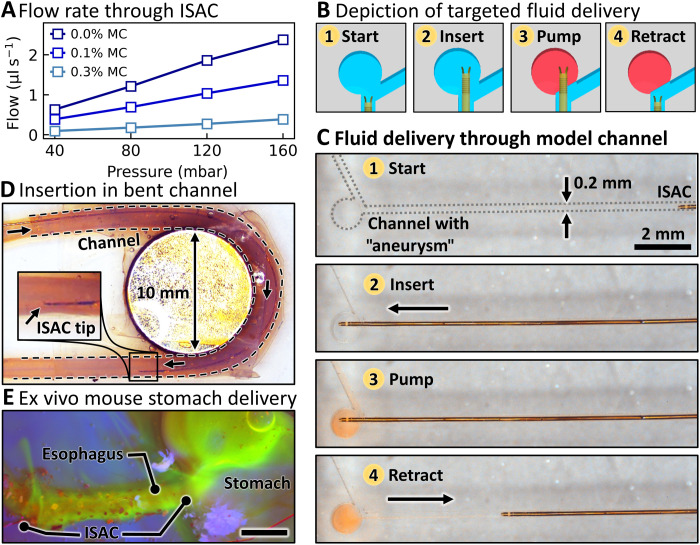
Characterization and demonstration of fluidic transport through ISACs. (**A**) Flow rate of liquids with different viscosities through an ISAC at various pressures. (**B**) Schematic depiction of the targeted delivery of a liquid payload. (**C**) Micrograph series showing the insertion of an ISAC through a model channel and subsequent delivery of a dyed model fluid into a target cavity (“aneurysm”). (**D**) Micrograph displaying a curved channel with ø = 0.9 mm into which an ISAC was inserted. (**E**) Micrograph of an ex vivo mouse stomach under blue light. An ISAC was inserted through the esophagus and delivered fluorescent dye into the forestomach. Scale bar, 5 mm. Photo credit: Boris Rivkin, Institute for Integrative Nanoscience, Leibniz IFW Dresden.

Here, we introduce a microcatheter that features integrated microelectronics embedded within the catheter wall (Fig. 1A). Figure 1B highlights its functionalities and various application scenarios. The integrated self-assembled catheter (ISAC) is connected to a microfluidic system that allows us to deliver a liquid payload in a targeted manner, e.g., to fill and seal a blood vessel defect, e.g., an aneurysm (Fig. 1B, 1). The catheter tip is equipped with a manipulator comprising four opposing digits, which allows the execution of micromanipulation tasks. A potential application could be the removal of clots out of blood vessels (Fig. 1B, 2). The demonstrated devices integrate magnetic sensors to monitor external magnetic fields and thereby enable position tracking and navigation (Fig. 1B, 3). In the application scenario depicted in Fig. 1A, the ISAC is deployed from a regular microcatheter, which serves as a host and accommodates electrical and fluidic connections. ISACs present an alternative fabrication paradigm for catheter manufacturing that relies on wafer-scale, microelectronic manufacturing processes and modern techniques for three-dimensional (3D) self-assembly of patterned thin films (*26*). This approach enables the simultaneous realization of the 3D geometry of the ISAC and the integration of electronic functionalities. Previously available electronically integrated catheters are larger than clinical microcatheters. Clinical instruments for, e.g., neurovascular interventions have diameters as small as 450 μm (1.35 Fr, 3 Fr = 3 French = 1 mm) (*27*). The ISAC technology creates devices with diameters between 50 μm up to 1 mm (*28*). In this way, the gap between electronically enhanced tools and the size requirements of vascular interventions in submillimeter anatomies can be bridged. This work focuses on ISACs with diameters around 120 μm as displayed in the micrograph in Fig. 1C. These small integrated catheters can lead to interesting applications such as surgical interventions inside, and drug delivery into, smaller and more delicate vessels (e.g., blood vesicles, female reproductive tract, ureter, and pancreatic duct) (*29*), point-of-care physiological and chemical sensing close to sites of interest or as minimally invasive neuronal or cardiac implants for electrical monitoring (*30*). In particular, for long-term sensing applications, the small diameter of ISACs can be beneficial, as it is expected to have no detrimental effect on the flow of blood in vessels with diameters greater than, e.g., 0.5 mm (*31*).

The fabrication strategy relies on a shapeable polymer stack, which has been reported previously (*32*, *33*). This material system is processed as a planar structure comprising ultrathin polymer layers and integrated electronic components and then self-assembles into microscopically thin “Swiss-roll” tubes. This technology has already led to the demonstration of numerous electronic devices that benefit from the geometry transformation. 3D energy storage devices have achieved higher energy densities through the markedly reduced footprint of rolled-up architectures (*34*, *35*). The polymer stack’s capability to reshape and reorient functional metal structures was key in realizing helical microantennas (*36*), transformers (*37*), and 3D magnetic encoders (*38*). More recently, self-rolling tubes have been explored as electronically functional channels in microfluidic systems, e.g., nuclear magnetic resonance chips (*39*), impedimetric microfluidic sensors (*40*), and biosensors (*41*). The shapeable polymer stacks have further been proven useful in devices that have to geometrically comply with other objects, such as cuff implants that wrap around “nerve bundles” in situ (*28*). Biohybrid micromotors were also realized with polymer scaffolds for sperm cell guidance and release upon a temperature change (*42*). Moreover, self-assembled polymeric structures served as reaction vessel and platform for electronic components to control the propulsion of microelectronic swimming robots by harvesting energy from external electromagnetic fields (*43*).

This work builds on the preceding development of rolled-up microelectronics to report an integrated catheter with multiple functionalities and a uniquely small diameter. Taking advantage of the multiple windings offered by the Swiss-roll architecture, a higher surface area is available to accommodate electronic components that are processed by state-of-the-art micropatterning techniques. A combination of subtractive and additive manufacturing techniques is used to equip ISACs with conductive polymer actuators that can be used for manipulation of microscopic and sensitive objects. The monolithically processed integrated magnetic sensor in combination with a purposefully developed navigation strategy enables position tracking of ISACs with a high resolution below 0.1 mm. This tracking approach does not rely on harmful radiation or contrast agents and is readily applicable in deep tissue and under dense materials, such as skull bones.

## RESULTS

### Wafer-scale fabrication and self-assembly

**Fig. 4.**
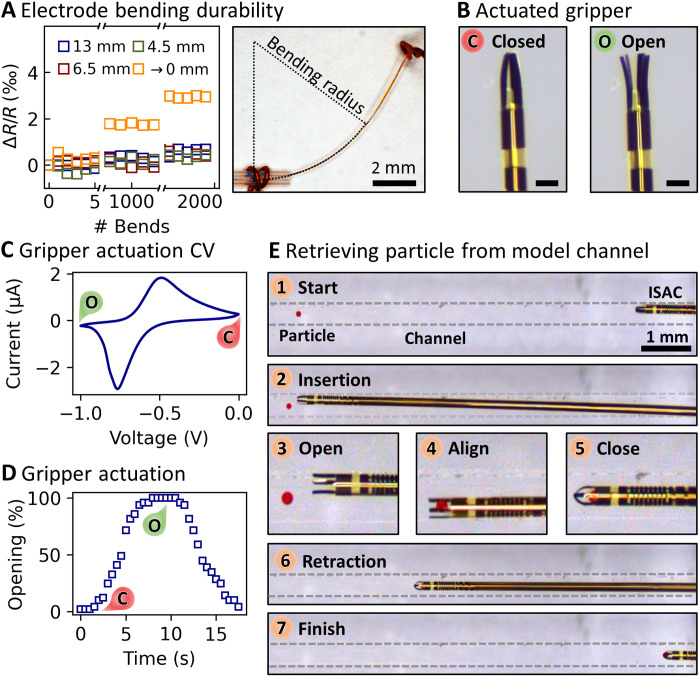
Assessment of electrode stability and demonstration of conductive polymer–actuated integrated manipulator. (**A**) An ISAC was bent with specific curvatures 2000 times each. The resistance change was recorded after each bending cycle. (**B**) Micrograph showing the integrated manipulator at the ISAC tip in its closed and open state. Scale bars, 100 μm. (**C**) Typical cyclic voltammetry (CV) curve of the conductive polymer actuated manipulator digits. The manipulator opens at reduction peak and closes at the oxidation peak. (**D**) Time-dependent opening and closing performance of an ISAC manipulator as obtained from microscope images. The manipulator fully opens within 5 s and closes within 6 s at a bias sweep rate of 100 mV s^−1^. (**E**) Micrograph series showing the retrieval of a 100-μm polystyrene particle out of a 0.4-mm-wide channel using the ISAC manipulator. Photo credit: Boris Rivkin, Institute for Integrative Nanoscience, Leibniz IFW Dresden.

**Fig. 5.**
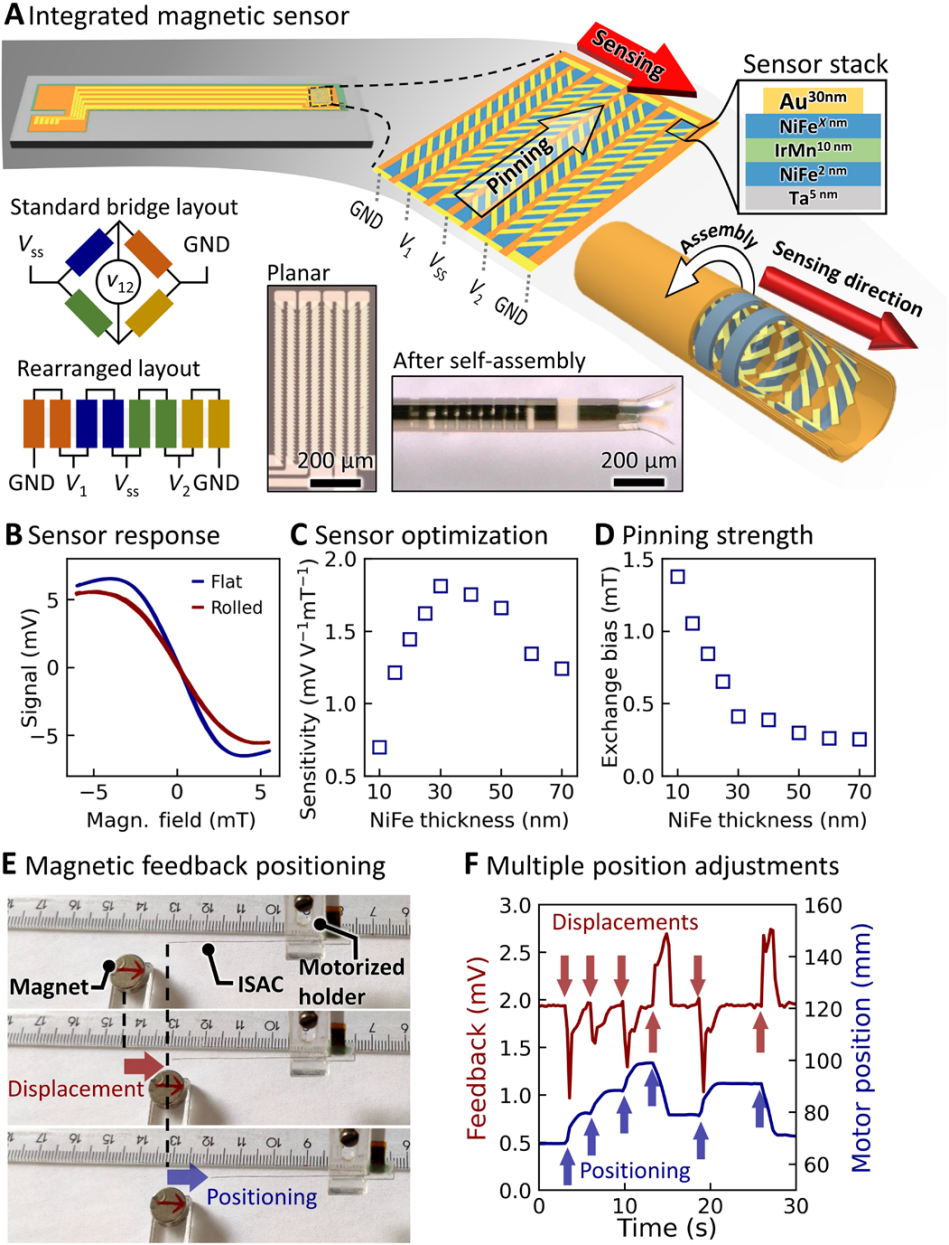
Development and characterization of integrated AMR sensors. (**A**) Schematic depiction of an AMR sensor bridge at the tip of an ISAC in the flat state and after self-assembly. The exact magnetic layer stack and the rearranged Wheatstone bridge circuit are displayed; micrographs of the sensor before and after self-assembly are included. GND, electrical ‘ground’. (**B**) Typical signal of an integrated AMR sensor during a magnetic field sweep. The same device was characterized before and after self-assembly with 1-V bias voltage. (**C**) AMR sensor sensitivity depending on the thickness of the topmost NiFe layer. (**D**) The exchange bias of AMR sensor stacks was assessed with magneto-optic Kerr magnetometry. (**E**) Real-time, automatic feedback-driven positioning of an ISAC. After a displacement of the reference magnet, a control algorithm readjusts the motor position to maintain a constant distance. (**F**) A series of reference magnet displacements and subsequent motor position adjustments. Photo credit: Boris Rivkin, Institute for Integrative Nanoscience, Leibniz IFW Dresden.

In this work, the fabrication of ISACs is similar to the devices highlighted above; details can be found in Materials and Methods. Briefly, a self-rolling polymer bilayer is photopatterned on top of a metal-organic sacrificial layer (SL) to make a shapeable polymer stack (Fig. 2A, 1). The thickness ratio of the constituent layers determines the diameter of the final ISAC (*40*). On top of the shapeable polymer stack, microelectronic components such as electrodes and sensors are fabricated through the standard microscale processing techniques (Fig. 2A, 2) and insulated with an ultrathin and stable metal-oxide film and subsequent polymer layer (Fig. 2A, 3). The insulating layers were patterned in a way to expose Au electrodes for the deposition of actuators and pads for electrical connections. Substrates carrying eight ISACS (image in fig. S1) were diced; devices were electrically connected, and the manipulator digits were electrochemically coated with the conductive polymer polypyrrole (PPy) (*44*, *45*). Then, the SL was selectively removed in a wet chemical process to initiate the catheter self-assembly into a rolled-up tube (Fig. 2A, 4). The shapeable polymer stack is pH sensitive during the assembly, and the tube diameter can be adjusted through pH regulation. After reaching their final shape (Fig. 2A, 5), ISACs are removed from the solution, completely dried, and subsequently rinsed with deionized (DI) water and phosphate-buffered saline (PBS) solution. The drying step is important, as it prevents future unrolling and permanently defines the diameter of the ISACs.

The self-assembly into rolled-up tubes is of key importance for ISACs for multiple reasons: (i) The channel that enables fluidic transport is naturally formed during this step. (ii) The initially parallel actuated digits are reoriented to face each other pairwise, thereby forming the manipulator and enabling enhanced grasping. (iii) The sensing elements of the magnetic sensor are transformed from stripes to spiral-shaped “rings,” which is crucial for their correct functioning. (iv) The entire ultraflexible device gains structural integrity once it transforms into the 3D tube, which is necessary to handle the final device in its freestanding form.

### Features and design considerations

The polymer stack and metallic components that constitute the ISAC before self-assembly are displayed in Fig. 2B. The entire device has a length of just under 50 mm (Fig. 2B, 1). At the distal end, four stripes of gold electrodes (width, 30 μm; length, 200 μm; micrographs before and after PPy deposition in fig. S2), which are supported by underlying polyimide (PI) and plated with PPy (Fig. 2B, 2; details in Materials and Methods), are positioned with a pitch of 90 μm to result in opposing digits for catheter diameters between 100 and 120 μm. The second electronic component of the ISAC is a magnetic sensor, located at the distal end as well (Fig. 2B, 2). Thin PI stripes (“anchors”; Fig. 2B, 3) extend from the shapeable polymer stack, over the SL, and connect to the glass substrate. They enforce the desired rolling direction (starting from left in Fig. 2B) and prevent a premature release of the device from the substrate during the self-assembly process. Metal traces (Fig. 2B, 3) electrically connect the magnetic sensor and actuators to five contact pads at the proximal end (Fig. 2B, 4). The metal traces are distributed over the entire width of the polymer stack, and each of them is subdivided into three stripes to minimize the effect on the self-assembly process. The electrical contact area is an appendix to the tubular main part of the ISAC and remains flat during the self-assembly as displayed in Fig. 2A (5). The proximal tip of the self-rolling catheter is used for fluidic connections [“rolling area” in Fig. 2B (4)].

The self-assembly of ISACs is driven by a swelling hydrogel (HG) that is located beneath the reinforcing PI layer. Its photolithographically defined pattern guides the rolling process and defines the mechanical properties. The HG features gaps that regulate the total strain and are tapered into trapezoidal shapes (fig. S3) in such a way that outside windings experience more strain compared to inside windings. This gradient reduces the distance between windings and improves device integrity. ISACs reported in this work have diameters of around 120 μm and three windings (Fig. 2C, 1), which proved as a decent compromise between stability and flexibility. A custom designed flex cable (thickness, 100 μm; length, 100 mm) with six electrodes is attached already before self-assembly (Fig. 2C, 2). A microfluidic tube (inner diameter, 0.3 mm; outer diameter, 0.7 mm) is installed at the free-standing proximal end of the ISAC and sealed with a two-component medical adhesive to establish a microfluidic connection. The ISAC and its connections are mechanically robust and can be handled manually (Fig. 2D) both in air and aqueous environments.

### Characterization of fluidic transport

ISACs with a fluidic connection are readily available for targeted delivery of liquid payloads. The flow through an ISAC was characterized using a pressure pump and liquids with different viscosities. The pump was adjusted to supply pressures between 40 and 160 mbar to a fluid reservoir, and the volume of transported fluid per unit time was measured (Fig. 3A). The viscosity of model fluids was adjusted by adding different concentrations of methylcellulose (MC; details in Materials and Methods). As expected from Hagen-Poiseuille’s law, the volumetric flow rate increases linearly with pressure but decreases for higher viscosity fluids, resulting in flow rates of 14.6, 8.1, and 2.4 nl s^−1^ mbar^−1^ for fluids with 0.0, 0.1, and 0.3% MC content, respectively. This measurement demonstrates the possibility to precisely control the amount of transported fluid through pressure and time with microliter precision. The viscosity of the studied model fluids can be estimated on the basis of available literature (η_0.0%_ ≈ 1 mPa·s, η_0.1%_ ≈ 2 mPa·s, and η_0.3%_ ≈ 5 mPa·s) (*46*). The study with pure water and viscous fluids hints at the wide range of potential applications, for instance, the harvesting of body fluids (blood, urine, oviduct fluid, bilis, gastrointestinal fluid, etc.) during liquid biopsy or the delivery of various drugs.

A key functionality of ISACs is the targeted delivery of fluids, which can be divided into four stages as depicted in Fig. 3B: Starting from an initial position outside the target area (1), the catheter is inserted (2) and the fluid is pumped into the cavity (3). Then, the catheter is retracted (4) after successful administration of the desired volume. A model channel (0.2 mm wide, milled into polymethyl methacrylate) with a round “defect” that mimics an aneurysm was used to demonstrate targeted delivery with an ISAC (Fig. 3C). Aneurysms are defects of blood vessels that can manifest as an outward bulging. Rupturing aneurysms can be lethal and are therefore treated through coil (*27*) or fluid (*47*) embolization, which both require catheters to be maneuvered close to or into the defect. In this study, the diameter of the artificial aneurysm is 1 mm with a narrow opening of only 0.6 mm. This particular aneurysm geometry is of interest since commercial catheters, due to their sizes generally larger than Ø = 450 μm, might not be suitable for the desired interventions (*29*). To demonstrate the targeted fluid delivery (Fig. 3C), we manually inserted the ISAC into the artificial microchannel and moved toward the target site, starting from a distance of about 10 mm (1), and inserted it into the defect (2). A dyed fluid, representing, e.g., a liquid embolic material (*47*), was injected through fluidic tubing and the ISAC into the target site (*3*) for 5 s. Once the injection was completed, the ISAC was retracted out of the defect. The administration was carried out manually, and thus, some liquid creeped out of the aneurysm and into the vessel due to the imprecise pressure control. Suction was applied to remove the small amount of excess dyed fluid, effectively demonstrating the capability of ISACs to collect liquid samples. Last, the ISAC was withdrawn completely out of the channel (4). The entire process can be found in movie S1.

The ISAC remained completely straight throughout the demonstrated model intervention. In a realistic environment, ISACs should be able to deform without structurally degrading in the process. This is particularly crucial when ISACs have to comply to the intricate channels with different bending angles present in the human anatomy. To assess shape adaptability, we prepared a curved channel (6-mm curvature radius, 180°) from a microfluidic tubing (ø = 0.9 mm) and manually inserted an ISAC multiple times (Fig. 3D). As can be seen in movie S2, the device seamlessly adjusts to the channel geometry without buckling, bending by 180° consistently and repeatedly. Fluid transport tests at constant pressure were repeated three times with the same ISAC in a straight and curved shape. The flow rate was hardly affected even when bent to the 6-mm curvature radius (219.0 ± 2.0 μl straight versus 218.7 ± 0.6 μl curved). In addition, it is important to verify that repeated bending does not unroll or damage the catheter. The mechanical durability was assessed in a stress test as shown in movie S2 as well, where an ISAC was kinked repeatedly by 30°, 60°, and 80°. Although kinking and buckling are generally not desired deformations during catheter interventions, they can occur during harsh handling and should not directly lead to catastrophic failure, such as breaking, tearing, or leakage. In this experiment, ISACs were intentionally kinked to maximize strain and emulate harsh operation conditions. For each deflection angle, an ISAC was bent 2000 times at the same spot. The mechanical integrity was regularly tested by running a dyed fluid through the catheter. For all bending angles, no leakage was observed at the bending sites even after 2000 deformation cycles, confirming the structural integrity of these devices.

Last, the fluidic delivery capabilities of ISACs were tested in an ex vivo organ that was retrieved from a mouse model to represent a more realistic environment. ISACs are envisioned for applications in diverse physiological environments, including the vascular systems, the female reproductive organs, and the intestinal tract. Regarding the latter, the esophagus and stomach of mouse models can adequately serve as testing environment while offering the small target lumen size of below 1 mm. An exemplary ex vivo organ is shown in fig. S4. An ISAC was manually introduced through the esophagus into the forestomach (movie S3), and a fluorescent dye was pumped into the lumen. The image in Fig. 3E shows the final scene under blue light. The dye accumulated in the upper part of the two-part rodent stomach and distributed into the esophagus as well. The ISAC did not remain completely straight during the insertion but adjusted its shape to comply with the vessel geometry. Future applications will require ISACs with even enhanced flexibility and active steering. This development will be subject to future studies.

### Reliability of electronic connections

ISACs rely on electronic leads that connect the manipulator and magnetic sensor to outside control and readout electronics. As catheters are generally subjected to repeated bending, the stability of connecting electrodes is critically important for reliable device operation. The durability of the electronic leads was assessed with an automated setup. As shown in the micrograph in Fig. 4A, ISACs were fixated, and the tip was actuated using a linear motor. The ISAC was bent to a desired curvature and then allowed to relax back to its initial straight shape. This process was repeated 2000 times, and the resistance (about 2 kilohms) of an electrode leading from the catheter base to its tip, and back, was measured in a two-wire configuration using a constant voltage and a series resistor (100 ohms). Various curvatures were applied, including mild bending with a curvature radius of 13 mm and kinking, where the entire curvature focused on a single point (radius → 0 mm) as displayed in fig. S5. Figure 4A shows the evolution of the normalized electrode resistance. It barely degrades for bending radii of 13.0, 6.5, and 4.5 mm, increasing by less than 1 per mil (‰). A more pronounced trend occurs when the ISAC is intentionally kinked sharply at a single point, mimicking unintended mishandling. This harsh deformation raises the electrode resistance by 2 and 4‰ after 1000 and 2000 bending cycles, respectively. The degradation might be attributed to the formation of microscopic cracks inside the electrodes (*48*). Hence, the durability could be increased with dedicated electrode shapes, e.g., meander shapes (*49*), as they are commonly used in flexible and stretchable electronics. Given these extremely harsh testing conditions, however, electrode stability does not appear to be a limiting factor for the presented devices.

### Digits with conductive polymer actuators for micromanipulation

Common catheter interventions include manipulation tasks such as implant installation, biological tissue introduction, biopsies, or retrieval of occluding objects, e.g., a blood clot. Using commercial catheters, these tasks usually require additional tools that are introduced through the catheter to reach the site of interest. In contrast, ISACs directly integrate a manipulator tool to carry out these tasks. Located at the ISAC tip, four opposing digits (Fig. 4B) can open and close to grasp and manipulate microscopic objects. The digits are actuated by a thin film of the conductive polymer PPy (*45*, *50*), which has been previously demonstrated in manipulation tasks (*50*–*52*). The actuation mechanism relies on the swelling and shrinking of PPy, which translates into a bending movement. The volume change is triggered through the electrochemically guided influx or repulsion of hydrated ions (e.g., Na^+^) from the surrounding fluid. Compared to the microfabrication of alternative stimuli responsive polymers, the deposition of PPy is easily integrated into common manufacturing workflows, enabling heterogeneous integration (*53*). PPy actuators require only low-voltage electrical biases and offer extremely fast and accurate shape control (*44*). Moreover, PPy naturally operates in aqueous environments, including various body fluids, and is widely regarded as nontoxic and overall biocompatible (*54*). Figure 4C shows a typical redox cycle of the ISAC manipulator. The digits are closed in the steady state at 0 V (versus Ag/AgCl). When sweeping toward a negative bias voltage (100 mV s^−1^), a negative current that peaks at −3 μA at −0.77 V (reduction peak) indicates the inflow of electrons into the PPy. This charge displacement is counterbalanced through the influx of positive ions from the electrolyte, which leads to the swelling of PPy and opens the manipulator. Sweeping the bias voltage back to 0 V has the opposite effect, and the manipulator closes again (oxidation peak at −0.49 V). Microscope images were used to measure the distance between two-digit tips to evaluate the opening and closing behavior of the manipulator quantitatively. Figure 4D displays one opening and closing cycle, where 0% opening refers to the initially touching digit tips and 100% when the manipulator reaches maximum opening. Opening the manipulator requires about 5 s with a bias scan speed of 100 mV s^−1^, and it takes another 6 s to fully close the manipulator. Note that this time is defined by the scan speed and actuator stacks of PI/Au/PPy were shown to fully reshape in less than a second (*44*). On the basis of previous reports on closely related actuated devices (*44*, *51*), the actuator force of ISAC manipulators can be estimated to be about 1 mN per actuator (details in Materials and Methods). The effective grasping force could, however, reduce if the manipulator digits misalign. This effect is summarized in fig. S6, indicating that 95% of grasping force is maintained if the rolling diameter varies by ±10%. If required, the diameter of the catheter can be more precisely controlled by introducing diameter-defining structures (*39*).

These soft actuators are ideally suited to handle delicate tissues. For instance, a typical task could be to retrieve an oocyte from a fallopian tube in the female reproductive tract. An ISAC was used to carry out a model intervention using an artificial channel [0.4 mm milled in poly(methyl methacrylate)] and a polystyrene microparticle (Ø = 100 μm). Figure 4E contains a series of micrographs showing this process, which can also be seen in full in movie S4. An ISAC was introduced into the channel (1) and moved toward the particle (2). Starting from the initially closed state, the manipulator was opened (3), aligned with the particle (4), and closed again (5). With the particle firmly secured by the manipulator, the ISAC was retracted and pulled out of the channel (6 and 7). The entire process was carried out manually and required constant optical feedback. The position of the target and the ISAC were monitored through a stereo microscope. However, direct optical observation of an ISAC will not be possible in most envisioned application scenarios within the human body.

### Development and application of an integrated magnetic sensor

State-of-the-art catheter interventions commonly rely on fluoroscopy imaging to monitor the propagation of the catheter. Monitoring through fluoroscopy requires repeated scans with ionizing radiation (x-rays), which is harmful both for the patient and the medical practitioner standing nearby to guide the catheter. Therefore, the navigation of medical tools using only on-board electronic sensors is a highly sought-after functionality (*55*). One established approach that relies on the probing of reference alternating (AC) or static (DC) electromagnetic fields, and more specifically their gradients, became known as magnetic or electromagnetic tracking (EMT) (*22*, *56*). Magnetic sensors such as pick-up coils or gradiometers were attached or introduced into tools such as biopsy needles, endoscopes, or catheters. In combination with an external field generator, medical tools are tracked within a patient, without the requirement for a direct line of sight and without harmful radiation or contrast agents. However, the size of the applied sensing elements remains prohibitive, and EMT is mostly used to monitor relatively big and stiff tools (biopsy needles and endoscope) and catheters that are used in the biggest blood vessels and heart chambers (*57*). A microcatheter that is highly flexible, yet offers tracking functionalities similar to those of EMT, would be a valuable addition to the toolkit of medical practitioners. Such a tool could extend the reach of fluoroscopy-less catheterization into areas with the smallest and most intricate vessel systems, such as the cerebrovascular system within the brain. This work reports, to our best knowledge, the first microcatheter with a monolithically integrated magnetic sensor. Moreover, it provides a strategy for the tracking of ISACs and evaluates said strategy experimentally.

Different magnetic sensors based on shapeable rolled-up polymer stacks have previously been reported, namely, antennas (*36*), giant magnetoimpedance sensors (*32*), and giant magnetoresistance sensors (*38*). The selection of an appropriate sensor type for tracking ISACs should take various aspects into account: (i) Navigation should rely on harmless low-intensity magnetic fields at low frequencies; therefore, sensors that require a saturation field were disregarded. (ii) Induction-based approaches impose requirements on the size of the involved antenna or pick-up coil, which are incompatible with the small target size and geometry of ISACs and were therefore also excluded. (iii) Since ISACs are rotationally symmetric by design, the sensor signal should not be affected by the rotation of ISACs around their long axis. Hence, the sensors should enable tracking with 5 degrees of freedom (DOFs), i.e., parallel displacement in three directions and rotation around the two axes that are orthogonal to the ISAC.

Ultimately, anisotropic magnetoresistance (AMR) sensors were chosen, as they can detect weak magnetic fields, e.g., the earth magnetic field (25 to 65 μT), and are useful as electronic compasses or for object localization tasks in a variety of industrial (*58*, *59*) and scientific (*60*–*62*) applications. In this work, an AMR sensor is integrated close to the ISAC tip as displayed in Fig. 5A. The sensor consists of eight parallel soft-magnetic NiFe stripes (length, 600 μm; width, 25 μm), which pairwise form the four arms of a Wheatstone bridge. Additional NiFe stripes can be added to increase the sensitivity at the expense of increased size. AMR sensors require a steady-state magnetization anisotropy that is commonly provided via an anisotropic shape (*60*), an external bias field (*63*), or an additional magnetic pinning layer, which induces an exchange bias (*61*). Here, an exchange bias is introduced by a pinning layer of IrMn underneath the NiFe, which pins the steady-state magnetization of the AMR stripes along their long axis (details in Materials and Methods). The AMR sensor is finalized with 5-μm-wide Au stripes, the so-called barber pole stripes. Since Au has a higher conductivity than NiFe, the stripes redirect the current onto a zigzag path through the magnetic stack, as the current flow will generally follow the path of least resistance. The stripes are arranged such that the current flows through the NiFe layers are effectively orthogonal for adjacent arms of the Wheatstone bridge. This common technique has been extensively discussed in previous literature (*64*, *65*). The magnetic stripes are oriented orthogonally to the catheter axis, such that they roll up to form rings during the self-assembly (Fig. 5A). After the self-assembly of the ISAC, the resulting sensor is sensitive to the component of a magnetic field that coincides with the catheter’s long axis and is insensitive to all components that are orthogonal to it because of its geometry and the integrated steady-state magnetization. Micrographs of the integrated magnetic sensor are displayed before and after self-assembly in Fig. 5A. Integrated AMR sensors were characterized using a Helmholtz coil setup, which generated a homogeneous magnetic field. Figure 5B displays typical sensor output curves for field sweeps in the range of ±5 mT for one device before and after self-assembly. Both curves have a linear regime around zero field, which then saturates at about 4 mT in the presented case. The curves display little noise and no substantial hysteresis. The minor deviation between the outputs of the planar and rolled-up sensor originates most likely from the additional strain, which influences the magnetic properties of NiFe through magnetomechanical interactions (*66*). The integrated AMR sensor was optimized to offer high sensitivity for very small magnetic fields. Here, the dominant device parameter concerning sensitivity is the thickness of the top NiFe layer. The optimal thickness was determined by a series of devices with NiFe layer thicknesses ranging from 10 to 60 nm. The sensors were characterized with bias field sweeps, and their sensitivity was derived from the slope of the sensor output around zero field. In addition, the pinning strength (exchange bias) of the magnetic stack was determined through Kerr magnetometry (*67*) to gain an improved understanding of the material system. Figure 5C summarizes the sensitivity measurements, while curves for each thickness are shown in fig. S7. An optimum occurs for devices with a NiFe thickness of 30 nm, yielding a sensitivity of 1.81 mV V^−1^ mT^−1^. This compares well with the performance of similar devices despite being, to our best knowledge, the first rolled-up AMR sensor and therefore substantially smaller than previously reported sensors (*60*, *65*). The relationship between the NiFe layer thickness and sensor sensitivity is readily explained using the optomagnetic Kerr measurement of the exchange bias as shown in Fig. 5D. The pinning strength relates antiproportionally to the NiFe layer thickness. It is highest for the thinnest NiFe layer, rapidly decreases at first with increasing layer thickness until around 30 to 40 nm, from where it only changes a little. Similar behavior was previously reported for related material systems (*61*). A higher exchange bias increases the measurement range of an AMR sensor but reduces its sensitivity. Therefore, the weaker pinning for layers with thicknesses around and above 30 nm are advantageous compared to thinner layers. The decline in sensitivity of sensors with NiFe thicknesses of >30 nm (Fig. 5C) is related to the current distribution through the magnetic stack. In summary, the study of the electrical and magnetic properties of the AMR sensor stack resulted in an optimized device with a 30-nm-thick NiFe layer.

A feedback-driven positioning test was carried out with an ISAC to verify the AMR sensor’s capacity to function in such applications: As displayed in Fig. 5E, an ISAC was mounted onto a holder that was placed on a linear motor stage. A magnet was positioned about 1 cm beneath and 1 cm in front of the ISAC, the response of the integrated sensor was then measured and used to define the set point. Afterward, the sensor signal was used as feedback in a proportional-integral-derivative (PID) control loop (*68*) to keep a constant distance between the magnet and the ISAC. Whenever the magnet was displaced, the software-controlled motor adjusted the position of the ISAC in real time with an update rate of 4 Hz. Figure 5F shows the feedback and output curves of a series of magnet displacements and subsequent motor position adjustments. The control loop was set up to maintain a sensor signal (feedback) at 1.94 mV (set point). Whenever the magnet was displaced, a rapid change of the feedback signal occurred as indicated with red arrows in Fig. 5F. The control loop adjusted the motor position quickly at first (blue arrows), slowed down when the ISAC was approaching the desired position, and held the catheter at a constant position once the feedback coincided with the set point. The controller managed to compensate 90% of a typical displacement (about 10 mm) within less than 1.5 s. This experiment is also shown in movie S5.

### Magnetic phase–encoded position tracking

Permanent magnets and static magnetic fields are suitable for simple positioning tasks as described above but cannot enable multidimensional navigation of medical tools using a single sensor. In contrast, alternating magnetic fields offer a reference frame that is suitable for this task, as demonstrated under laboratory (*22*, *69*) and clinical (*70*) conditions. The precise tracking of an ISAC requires a reference frame that is rich in information and can be detected with the integrated AMR sensor. Here, we first identify an attractive application field for ISACs and their navigation, define relevant experimental parameters and benchmarks, develop a tracking strategy, and verify it experimentally.

The most appealing application of ISAC navigation is the brain, where other tracking techniques that rely on light or ultrasound (US) are blocked by the skull. Classic EMT devices might be too bulky to maneuver through intricate blood vessel networks (*56*), and navigation thus solely relies on x-ray based imaging. For neurovascular microcatheter interventions, ISAC navigation should offer extremely high tracking precision (e.g., <<1 mm) within a limited working volume of around 20 cm across. Here, we develop such an alternative strategy for magnetic navigation, which enables higher tracking resolution within a smaller working area, compared to classical EMT. In this sense, our work intends to complement existing navigation techniques rather than directly compete with them.

To maintain the focus of this work, we only demonstrate the most elemental form of displacement tracking, 1D parallel displacement along a line. Consecutive works will extend the presented tracking strategy to include multidimensional displacement and rotation up to 5 DOFs, as outlined in Supplementary Text.

**Fig. 6.**
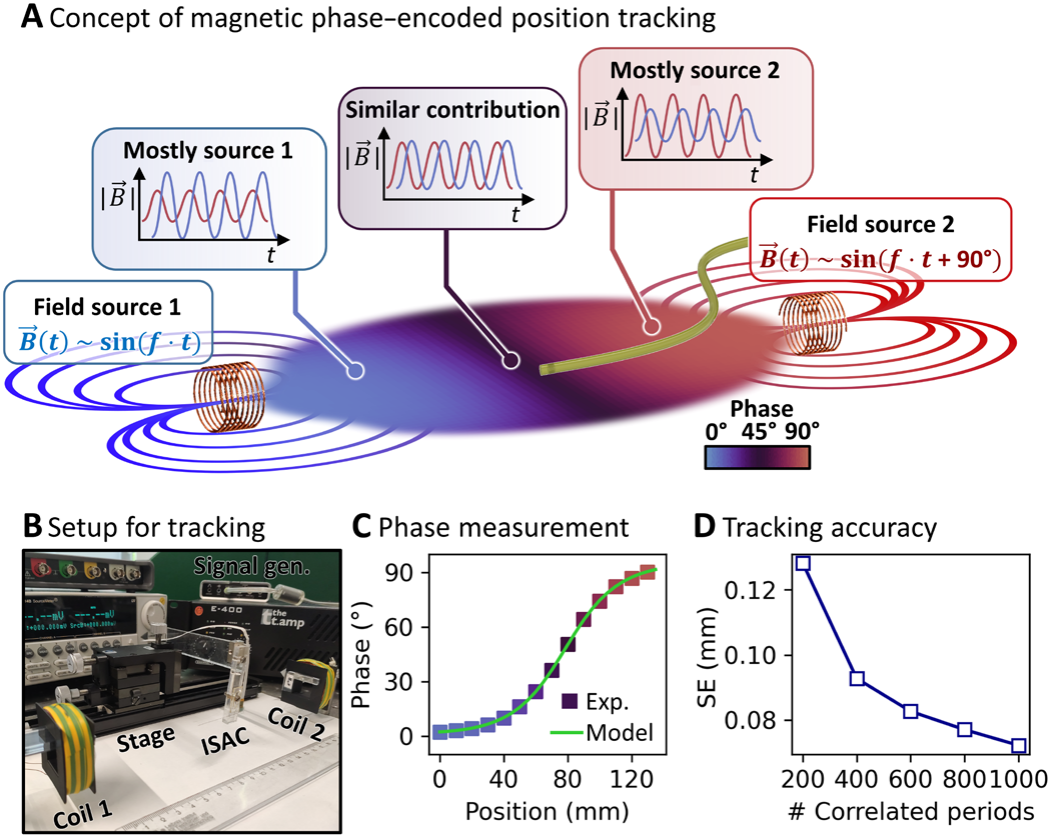
Concept and evaluation of magnetic phase–encoded position tracking. (**A**) Schematic depiction of the proposed navigation strategy. Two electromagnetic coils generate a magnetic field with a position-dependent phase, which can be detected by an ISAC and used for navigation. (**B**) Photograph of the experimental setup. An ISAC is mounted onto a motorized holder in between two electromagnetic coils. (**C**) The experimentally determined, position-dependent phase of the reference signal measured with an ISAC and the corresponding fitted model. (**D**) The precision of position tracking improves with longer signal length. Photo credit: Boris Rivkin, Institute for Integrative Nanoscience, Leibniz IFW Dresden.

Figure 6A illustrates the basic principle of magnetic phase–encoded navigation. Two electromagnetic coils are placed at opposite sides of the working volume. They receive alternating sinusoidal currents with the same amplitude and frequency but with a phase difference of 90°. Inside the working volume, the fields from both coils superimpose. The contribution of either coil toward the superimposed field strongly depends on the position within the working volume because the field strength decays ($|\vec{B}|$ ∼ *r*^−3^) with increasing distance from the emitting magnetic dipole. Consequently, the superimposed magnetic field has a unique, position-dependent phase, as indicated by the color map in Fig. 6A. When assessed along a straight line between both coils, the phase relation takes the shape of an arcus tangents between 0° and 90°, featuring two plateaus close to the coils and a steep phase gradient in the center. The derivation of the model in 1D and 3D can be found in Supplementary Text.

An experimental setup was designed to test the tracking strategy and assess its performance with ISACs. Two electromagnetic coils (750 windings, 20 mH) were manufactured using 3D printed frames and enameled copper wires. An image and impedance curves are displayed in fig. S8 (A and B). The coils were fixed on an optical table, facing each other at a distance of 20 cm (Fig. 6B) and supplied with sinusoidal current (1 A peak to peak, 100 to 1000 Hz). The magnetic field generated by a single coil decayed exponentially as displayed in fig. S8C, having harmless (*71*) field strengths of, e.g., 0.2 mT root mean square at 5-cm distance. An ISAC was attached onto a high-precision motorized linear stage and placed between the signal coils as shown in Fig. 6B. The AMR sensor signal was amplified and recorded with an oscilloscope (details in Materials and Methods; schematic and image of setup in fig. S9). The current through one of the coils was recorded and used as the reference signal.

To confirm the unambiguous position-phase relation, we moved an ISAC stepwise along a straight line in the center region between the two coils over a distance of 14 cm. At each step, the sensor signal was amplified, recorded, and processed. The phase relation was obtained by cross-correlating the sensor signal and the reference signal (coil supply current). Reference and sensor signals at the various positions, as well as the corresponding cross-correlates, are displayed in fig. S10. A phase shift between the obtained signal and the reference emerges as the sensor is moved through the working area. The cross-correlation of the reference and each sensor signal has a maximum that provides a numerical value for the phase shift. Figure 6C shows the position-phase relationship. It features the predicted behavior with the pronounced phase gradient in the center, which flattens toward both sides at 0° and 90°. The measured phase shifts are in good agreement with the fitted model and confirm the unique relation between the sensor signal and real position. Thus, the proposed model is suitable for the tracking of ISACs.

After establishing the general validity of the magnetic phase–encoded tracking, its precision requires closer investigation. Since the magnetic phase encodes a position, a high phase gradient enables high spatial resolution. There, small position changes will lead to substantial phase changes. The theoretically possible spatial resolution depends on two factors: (i) the gradient of the phase and (ii) the temporal accuracy with which the phase is measured by the readout electronics, namely, the analog-to-digital converter (ADC). Since the phase shift between the two signals is determined via cross-correlation, the smallest measurable phase shift is ΔΦ_min_ = 360° × *f*_Signal_ × *f*_ADC_^−1^, with the signal frequency *f*_signal_, the ADC sampling frequency *f*_ADC_, and a factor of 360° to convert points per period to points per degree. In simple terms, the phase shift measurement is more precise when more data points per signal period are obtained. On the basis of the measurement depicted in Fig. 6C, the phase of the reference field changes by δΦ = 60° over a distance of δ*x* = 50 mm. With the signal frequency of *f*_Signal_ = 100 Hz, the minimum spatial resolution is Δ*x*_min_ = δxδΦ×ΔΦmin = 30 μm with *f*_ADC_ = 1 MHz. This theoretical limit, however, assumes an ideal measurement, while real signals are heavily distorted by noise. Adverse distortions due to noise are averaged out through the cross-correlation of numerous signal periods. This computation is more reliable when more signal periods are included. To assess the real resolution of the tracking scheme, we used a static integrated AMR sensor to measure the magnetic reference field for 200 times. The phase shift was calculated using different numbers of signal periods from 200 to 1000. Histograms showing the entire measurement spreads depending on signal length can be found in fig. S11. The SDs of the phase measurements were converted to a position uncertainty using Δ*x*_min_ = 30 μm and summarized in Fig. 6D. The SE of the estimated position is about four times the theoretical resolution, about 0.13 mm, when only 200 signal periods are considered. The resolution improves to 0.09 and 0.08 mm when 400 and 600 periods are included, respectively. The benefit of additional signal length saturates beyond 600 periods, and a tracking SE of 72 μm is achieved when 1000 periods are cross-correlated. The apparent tradeoff between temporal performance and tracking accuracy should be noted, as improved accuracy requires longer signal acquisitioning times. The overall performance can be increased using higher frequency reference fields combined with higher ADC sampling rates to reduce the signal acquisitioning time. The choice of signal frequency, however, must be within the limits of available signal sources. Furthermore, cross-talk with readout electronics and biocompatibility and safety need to be considered (*71*).

The presented study of real tracking accuracy confirms the general applicability of magnetic phase–encoded tracking. Available navigation systems commonly advertise tracking precisions of about 0.5 to 1.5 mm in 3D and with 5 to 6 DOFs within typical working volumes of 0.5 m by 0.5 m by 0.5 m (*56*) and using relatively bulky sensing elements with typical sizes larger than 0.3 mm in diameter and longer than 5 mm (*72*). In contrast, ISACs rely on microsized integrated AMR sensors with 0.12 mm in diameter and only 0.35 mm in length to accomplish tracking with a high resolution smaller than 0.1 mm as demonstrated in 1D. This novel tracking scheme outperforms previously available systems in terms of precision and sensor size but at the expense of working volume. In practice, ISACs could complement available technologies for applications with high precision demands, small working volumes, and the requirement for highly flexible microcatheters. Possible applications could be the navigation toward and into small aneurysms or the minimally invasive readout of neural signals that can be recorded from blood vessels within the brain (*30*).

### Simultaneous tracking with magnetic phase–encoding and US

While navigation based on magnetic fields can provide precise location data, its applicability is limited if not combined with images of patient anatomies (*69*). In practice, images obtained with magnetic resonance imaging (MRI), computed tomography, or comparable techniques should serve as “maps” into which tracking data can be embedded. Here, we investigate the compatibility between ISACs and their magnetic phase–encoded tracking and US imaging. US is a well-established imaging approach to visualizing anatomic structures in soft biological tissues, for instance, in the abdominal or throat region. Since mechanically hard structures reflect US waves, the imaging of body parts that are surrounded by solid bones, such as the brain, is generally not feasible.

**Fig. 7.**
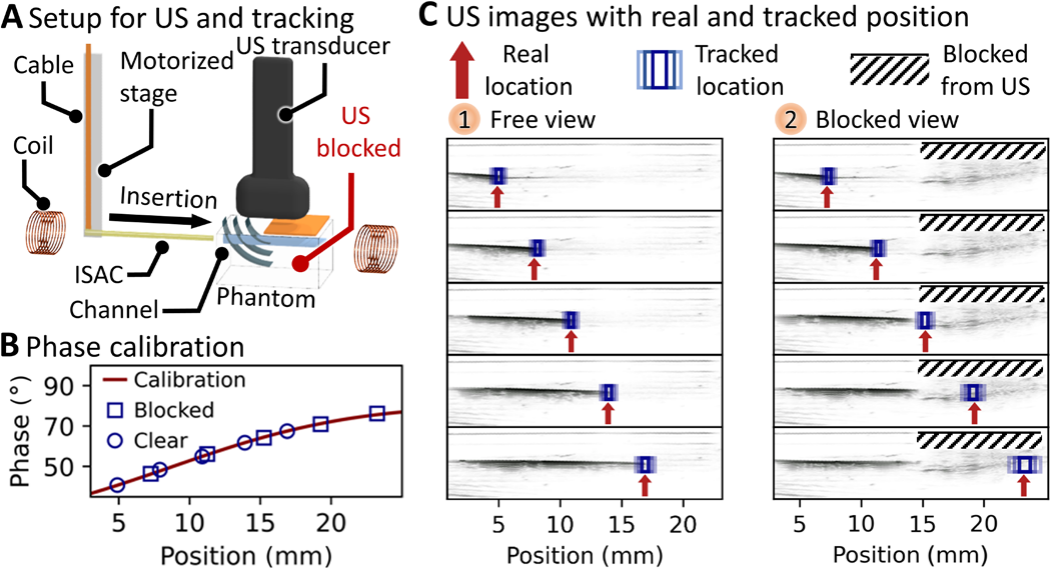
Simultaneous magnetic phase–encoded tracking and US observation. (**A**) Schematic of experimental setup. (**B**) The calibration curve and measured phases during simultaneous US and magnetic phase–encoded tracking. (**C**) US image series of an ISAC being inserted into an agar phantom. The real position of the ISAC tip is defined by the motorized holder. The tracked location is indicated with threefold boxes, indicating one, two, and three SDs. Photo credit: Boris Rivkin, Institute for Integrative Nanoscience, Leibniz IFW Dresden.

Simultaneous magnetic phase–encoded and US tracking of ISACs was carried out in a model environment as schematically depicted in Fig. 7A. Inside the working area for magnetic phase–encoded tracking, an agar phantom (40 mm by 35 mm by 20 mm) with a hollow channel (Ø = 0.7 mm) was immersed in a water tank. The phantom was oriented such that the channel coincided with the tracking axis connecting the two coils. A commercial US imaging system (FUJIFILM VisualSonics) was implemented; the transducer was positioned above and along the channel, and a precise linear motor stage was used to move the ISAC inside the phantom channel. The US images were recorded using a 256-element linear array US transducer at a central frequency of 21 MHz, and the signals were collected and reconstructed using the on-board postprocessing.

First, a calibration was carried out to relate measured phase shifts and real positions (details in Materials and Methods). The resulting calibration curve is displayed in Fig. 7B, and the constituent data can be found in fig. S12. Then, the US transducer was adjusted to monitor the catheter inside the phantom channel. ISACs have good US contrast due to the US reflecting at embedded metal electrodes. Movie S6 shows the continuous insertion and retraction as obtained by the US imaging. Simultaneous magnetic tracking and US observation were carried out as summarized in Fig. 7C (1). From top to bottom, the series of US images indicate the stepwise insertion of an ISAC (seen as dark horizontal structure) into the model channel. The red arrows in Fig. 7C indicate the real ISAC location as it is known from the precisely defined motorized stage position. The tip positions as seen in the US image are in good agreement with the real positions. Threefold blue boxes indicate one, two, and three confidence intervals within which the position is predicted by magnetic phase–encoded tracking. The tracked location shows excellent agreement with both US images and real positions, matching within one SD in four cases and within two SDs in one case.

Afterward, a dense object was placed on the agar phantom to partially prohibit US feedback, thus recreating the scenario of brain imaging where the skull bone tissue blocks the US waves. For the image series in Fig. 7C (2), copper foil was used to partially cover the field of view (right half) of the US transducer. This obstacle blocked US from imaging the region beneath and left a noisy blank area. Movie S6 shows a continuous ISAC insertion under these conditions where the catheter is well visible in the left half but cannot be observed after entering the blocked region. The ISAC was then stepwise inserted in the modified phantom. In the first and second panel of Fig. 7C (2), the tracked position agrees well both with the US and the real location within one SD. Starting from the third image, however, the catheter tip enters the blocked area and is not visible under US from there on. At the same time, the magnetic tracking reliably provides the ISAC tip location within one SD compared with the real position. Note that the width of the confidence intervals increases from the top to the bottom image. This occurs naturally because of the flattening of the phase-position calibration curve toward the edges of the working area and the consequent reduction of spatial resolution. The individual phase shifts used for position reconstruction in the image series are indicated in Fig. 7B.

This study demonstrates that ISACs can work in tandem with imaging techniques that provide anatomical details: While the US indicates the location of a hard object (here, the Cu foil), magnetic tracking reliably provides location data although the complementing technique cannot. Future minimally invasive catheter interventions could hugely benefit from preoperative high-resolution MRI scans with embedded real-time tracking data to navigate a surgeon precisely and reliably toward the target site, entirely without harmful radiation or contrast agents.

## DISCUSSION

In this work, we presented an electrically integrated microcatheter that is enabled by a wafer-scale self-assembly process. This fabrication strategy constitutes a novel paradigm for the manufacturing of biomedical tools and enables multifunctional catheters with microscopic sizes that are entirely unprecedented. The multiple windings of the Swiss-roll architecture substantially increase the usable surface area, allowing the integration of more electronic components and thus leading to a higher integration density. The embedded electronic leads substantially decrease the manufacturing complexity and device size. Last, the used fabrication techniques of photolithography and self-assembly intrinsically enable a high throughput by parallel manufacturing. The ISACs feature three key functionalities: fluid delivery, micromanipulation, and magnetic navigation.

ISACs were integrated into a microfluidic system to transport liquid payloads in a targeted and flow-controlled manner, both into model structures and an ex vivo organ. Further studies will need to assess whether a wide variety of medically relevant substances such as drugs and body fluids can be transported as well.

Mechanical and fluidic tests verified adequate stability of the polymer tubes and the embedded electrodes under bending. Compared to available robotic catheter systems that require stiff components, ISACs integrate only soft and flexible compounds. Although ISACs show already good flexibility, it is yet to be assessed whether they can comply with more complex and tortuous environments, such as different vessels. As of now, ISACs resemble nonreinforced, flow-directed microcatheters and thus could be useful in similar interventions such as the treatment of brain arteriovenous malformations (*73*). Commercial catheters generally rely on a stiffness gradient, and their flexibility increases toward the distal end to facilitate compliance with curving vessels. A similar feature can be introduced into ISACs through further adaptation of the polymer stack microstructure. Batch fabrication and wafer-scale self-assembly will allow cost-effective manufacturing of hygienic single-use instruments on a large scale. Potential manufacturing imperfections might lead to device-to-device variations, especially regarding the rolling diameter. Additional polymer structures can be incorporated onto the innermost winding (*39*) to precisely define the geometry of the rolled-up devices.

ISACs were equipped with PPy actuated digits to grasp and manipulate microscopic objects. PPy actuators offer advantageous properties such as fast and precise response, low power requirements, and biocompatibility. In addition, PPy microactuators can be operated in combination with integrated sensors, enabling feedback control to mechanically detect soft tissue and handle it carefully (*44*). Because of the relatively small blocking force of PPy actuators, the manipulator is mostly suited to handle soft and highly sensitive objects. Alternative manipulation strategies, for instance, through suction, will be required to move objects that are bigger or firmly attached to vessel walls.

This is, to our best knowledge, the first report of a self-assembled system integrated with electroactive polymer actuators and represents a path breaking contribution toward self-assembling microrobotic tools. The next objective should be the integration of numerous independent PPy actuators along the entire ISAC to achieve full shape control with multiple DOFs. Such a distributed set of actuators will introduce active steering to ISACs, similar to active guide wires, and ultimately allow us to maneuver even through intricate vessel networks. To this end, the polymer tube will be further microstructured with additional patterns such as those known from origami (*74*) to enable efficient deformability. A secondary challenge will be the accommodation of numerous components (sensors and actuators) and their electronic connections within limited available space. Our ISACs carried only two components and a total of five electronic connections. Future integrated catheters might comprise dozens of sensors and hundreds of individually addressable actuators, which would rapidly exceed the polymer stack’s capacity to accommodate electrodes for each individual component. Advanced logic circuits based on thin-film transistors (TFTs) such as active matrices and shift registers (*75*) will be required to address each component individually and achieve a higher integration density. A related system has been reported recently with indium gallium zinc oxide TFTs and PPy actuators (*53*).

AMR sensors were optimized and integrated into ISACs to enable magnetic field measurements for navigation. Despite the restrictions that were imposed by component integration, the sensor sensitivity compares well with similar reported devices and was successfully used in magnetic feedback–driven positioning using a permanent magnet reference.

Last, a novel tracking strategy based on the phase measurement of a temporally and spatially varying reference magnetic field was introduced. This work provides a proof-of-principle investigation, demonstrating a high tracking resolution of down to 72 μm under laboratory conditions. The implementation of tracking capabilities into a device as thin as a human hair was achieved using integrated AMR sensors. Compared to commercial magnetic sensors, integrated AMR sensors can be more compact, as they do not require extra packaging, internal conditioning circuits, and bonding wires and use multiple windings to efficiently accommodate magnetically sensitive material. A strategy toward 5 DOFs navigation in 3D is described in Supplementary Text and requires a higher number of source coil pairs (five or more) to provide enough reference dimensions and enable high precision within the entire working volume.

All incorporated materials that interface with the environment, i.e., HG, PI, and PPy, have been previously assessed through cytotoxicity tests (*28*, *76*, *77*), showing overall biocompatibility. However, future studies with ex vivo and especially living tissues are required. Studies with ex vivo tissues will provide opportunities to evaluate the mechanical performance of ISACs, while in vivo studies with animal models need to investigate physiological interactions such as acute irritation and toxicity, hemocompatibility, immunoresponse, and effects of actuator currents, and device handling, deployment, and degradation. ISACs might require surface coatings (*78*) or anticoagulation measures to further improve maneuverability and biocompatibility.

In conclusion, the presented study reports on a microcatheter with integrated electronic functionalities that can be fabricated on a wafer scale through the self-assembly of ultrathin polymer stacks. Specifically, we have demonstrated a Ø = 120-μm small catheter with multiple functionalities. Further integrated sensing capabilities will expand potential application scenarios, for instance, blood gas analysis, biomolecule detection, and physical parameter assessment (pH, temperature, and pressure). Last, ISACs will be equipped with a distributed network of microactuators, leading the way to monolithically integrated, microrobotic catheters.

## MATERIALS AND METHODS

### Preparation of substrates

Float glasses with dimension of 50 mm by 50 mm by 1 mm (D263T eco glass, SCHOTT AG, Mainz, Germany) were cleaned in a professional washer DS 500 (Steelco S.p.A., Riese Pio, Italy) and treated with oxygen plasma in a GIGAbatch 310M (PVA Metrology & Plasma Solutions GmbH, Wettenberg, Germany). Cleaned glasses were placed in a vacuum oven at 150°C for 2 hours together with 250 μl of 3-(trimethoxysilyl) propyl methacrylate (TMSPM, Sigma-Aldrich) to form a monolayer of TMSPM as adhesion promoting surface modification.

### Preparation of shapeable polymer platform

All chemicals were purchased from Sigma-Aldrich unless noted otherwise. Polymer solutions were synthesized following published procedures (*32*, *36*, *37*). The shapeable polymer platform comprises (from bottom to top) a lanthanum–acrylic acid complex–based SL, a swelling HG layer, and a reinforcing PI layer. All layers are prepared as follows: (i) A thin film is deposited by spin coating. (ii) Excess solvent is removed with a soft bake (SB) on a contact hot plate under a constant stream of nitrogen. (iii) The pattern of the layer is defined through ultraviolet lithography (EXP) using a MA6 mask aligner (SÜSS MicroTec SE). (iv) Unexposed excess material is removed in a development step (DEV). (v) The layers are finalized with a hard bake (HB) at 220°C. The SL was SB at 35°C for 10 min, EXP with 250 mJ cm^−2^, DEV in DI water for 30 s and rinsed in (1-methoxy-2-propyl) acetate (PMA), and HB at 200°C for 10 min. The SL layer thickness was 200 to 300 nm. HG was SB at 40°C for 10 min, EXP with 450 mJ cm^−2^, DEV in diethylene glycole monoethyl ether (DEGMEE) for 2.5 min and rinsed in PMA, and HB at 200°C for 10 min. The HG layer thickness was 500 to 600 nm. PI was SB at 50°C for 10 min; EXP with 600 mJ cm^−2^; DEV in a mixture of *N*-ethyl pyrrolidone, DEGMEE, and ethanol with a volume ratio of 4:2:1 for 3 min and rinsed in PMA; and HB at 200°C for 10 min. A total of two PI layers were deposited: one on top of the HG and a second on top of metal traces and sensors. Both PI layers had a thickness of 500 to 600 nm. The layer stack was engineered such that metal traces and sensors were located in or close to the neutral plane and therefore experience only little strain during rolling and bending.

### Deposition of AMR sensors and metal traces

In this work, exchange bias pinned AMR sensors stacks comprised Ta^0.5nm^/Ni_80_Fe_20_^2nm^/Ir_19_Mn_81_^10nm^/Ni_80_Fe_20_^30 nm^. Magnetic layer stacks were fabricated with a standard lift-off process using a high-resolution photoresist AZ 5214e (MicroChemicals GmbH, Ulm, Germany) that was photolithographically patterned. The magnetic stack was prepared by magnetron sputtering with a base pressure of 2.4 × 10^−6^ mbar and a 1.4 × 10^−3^ mbar Ar atmosphere during deposition inside a homogeneous magnetic field (25 mT). The devices were aligned with the field such that the magnetic stripes were magnetized parallel to their long axis. Then, the photoresist was dissolved in acetone to remove excess magnetic material. All further metal traces (sensor interconnects, barber pole stripes, and electrodes for actuator deposition) were patterned with a single lift-off step as above. A Ti^4nm^/Au^30nm^ was deposited by means of electron beam evaporation, and excess material was removed with acetone. Thin layers of Al_2_O_3_ (20 nm each) were deposited both before and after the metal layers to offer additional chemical and electrical insulation. Excess Al_2_O_3_ was removed via wet chemical etching at room temperature in a 2.7% solution of tetramethyl ammoniumhydroxid (20-min etch time) after the deposition of the topmost PI layer, which served as etching mask.

### Electropolymerization of actuators

Actuators based on the conductive polymer PPy were electrochemically deposited, conditioned, and operated following previously reported procedures (*44*). Briefly, the bare Au electrode were cleaned with acetone, 2-propanole, treated with oxygen plasma for 1 min, and connected electrically as working electrode in a three-electrode setup with a AgCl-plated Ag wire (Thermo Fischer Scientific) reference electrode and a Au-plated Cu rod as counter electrode. The three electrodes were submerged in an aqueous monomer solution [0.1 M pyrrole and 0.1 M sodium dodecylbenzene sulfonate (NaDBS)] and connected to a potentiostat/galvanostat (PGU20A, IPS, Germany). A constant voltage of 520 mV was applied for 27 s to deposit 2.4-μm-thick PPy layers. The deposition current increased slowly throughout the process from 3 to 4 μA and transported a total charge of 8.2 × 10^−8^ mA·s. The devices were rinsed with DI water after PPy deposition.

The force output of PPy actuator has been previously investigated with closely related devices (*44*). Moreover, it has been reported that the force that is generated by these curving actuators at their tips scales linearly with the actuator width and antiproportionally with their length (*51*). On the basis of these previous reports and the geometry of ISAC actuators (width, 30 μm; length, 200 μm), the force of the integrated manipulators can be estimated to be about 1 mN per actuator. The effective grasping force equals the digit force for perfectly aligned manipulators but reduces for misaligned digits. To estimate the effective force of misaligned digits (fig. S6), one first calculates the digit angle depending on the rolling diameter error to then compute the parallel force components of opposing digits.

### Self-assembly of the shapeable polymer stack into rolled-up tubes

The transformation of flat polymer layers into rolled-up catheters was carried out as follows: First, the SL was selectively removed in a mild HCl solution (2%_vol._) to release the shapeable polymer stack from the substrate. Devices were then transferred individually in a basic solution (NaOH in DI water) where the pH-sensitive HG expanded and reshaped the layer stack into a roll-up tube. The pH of the rolling solution was adjusted to achieve the desired tube diameter that was controlled in situ through a microscope, depending on the individual layer thicknesses and the structure of HG. For instance, pH 9.5 was required to properly roll a tube with 600 nm of HG and 1200 nm of PI, where HG patches were 5 mm long and had gaps of 0.16 mm. Once the catheter reached the desired shape, the substrate with the still attached device was removed from the rolling solution, and a layer of camphor was deposited immediately on the still wet devices. The deposition was carried out with a regular spray bottle and a solution of camphor in acetone and water (13:9:4 weight ratio). Bare rolled-up catheters may unroll while drying, so the camphor layer was applied to mechanically lock the still wet devices in shape and prevent unrolling. Water then evaporated through the camphor layer that subsequently sublimated within 6 hours to generate tightly rolled and stable tubes, which do not unroll in air or aqueous environments. Hence, prepared ISACs were rinsed with DI water and stored in air or PBS solution.

### Application of fluidic and electronic connections

Electronic connections were applied before PPy deposition. Custom-designed flexible cables (six Cu traces, 100-μm total thickness) were purchased from Multi Leiterplatten GmbH and hot press bonded to samples with a custom setup and anisotropically conducting tape.

Fluidic connections were established after self-assembly, drying, and rinsing of ISACs. Microfluidic tubing was manually put over the proximal tip of ISACs, and a drop of medical adhesive was applied to seal the tube connection. After curing, additional medical adhesive was applied to partially attach the flexible cable and fluidic tube. The free end of the tubing could then be connected to standard fluidics equipment.

### Measurement of fluid flow rate through ISAC

The fluid flow through ISACs was measured for aqueous solutions with different viscosities. MC was weighted into DI water (0.1 and 0.3% by mass) and stored at 8°C with repeated shaking until completely dissolved. A pressure pump (Biophysical Tools GmbH, Leipzig) was used to create a flow through the ISAC and its tubing connection. The fluid reservoir was weighted with a precision scale before and after a recorded pumping time (5 to 10 min depending on flow rate), and the mass difference was used to calculate the transported volume.

### Preparation of ex vivo mouse stomach

The retrieval of the esophagus and stomach is done first by first making an incision at the center bottom of the mouse and cut toward the top. There, because of the presence of the connective tissue and skin, an additional cut at the bottom of the abdominal wall is required, cutting up until feeling some resistance, as this area is covered by the rib cage. At this point, the organs of the digestive system are visible, and one can proceed to carefully cut and retrieve them for further experiments. Before insertion experiments, the intestine was removed from the stomach. The stomach and esophagus were then put in a PBS-filled petri dish and mounted on a rubber substrate using thin Cu wire. Residual stomach content was flushed out using a syringe and fine needle. Experiments were conducted under licenses nos. DD24.1-5131/394/78 (TVV74/2017) (Permission—Generating mouse models) and DD24.1-5131/461/1 (Permission—Biotechnologies).

### Measurement of exchange bias with Kerr magnetometry

A wide-field Kerr microscope setup (*67*), making use of the magneto-optical Kerr effect (MOKE) and equipped with an electromagnet, was applied to measure magnetic hysteresis loops. Magnetization was measured by applying the longitudinal Kerr effect with the magnetic field and magneto-optical sensitivity being aligned along the AMR stripe axis. Hysteresis loops were generated by plotting the image intensity of a selected image spot as a function of the applied magnetic field. To reduce the noise, the MOKE intensity of eight AMR stripes was measured, and eight image frames were averaged for each measurement. The exchange bias was deduced from the center of the MOKE hysteresis curves, which is displaced toward positive values of the applied field.

### Characterization of AMR sensors

AMR sensors were characterized in a homogeneous magnetic field, which was generated by two coils in a Helmholtz configuration and monitored with a calibrated Hall magnetic sensor. A custom-developed printed circuit board (PCB) with eight 32-bit differential ADC and eight 16-bit digital-to-analog converter (DAC) operating at 1 kHz was used to supply and measure the integrated AMR sensor, read out the reference hall sensor, and control the power supply of the Helmholtz setup. AMR sensors were placed in the center of the Helmholtz setup such that their sensitive axis aligned with the field direction. The field strength was swept from −12 to 12 mT and back to −12 mT with intervals of 0.1 mT. The AMR sensors were supplied with a constant bias that yielded a 1-V voltage drop at the sensor bridge, and 40 measurement points were recorded and averaged for each field strength.

### Position control with AMR sensor feedback

The abovementioned ADC-DAC-PCB was used to supply and read out the integrated AMR sensor during feedback-driven positioning. The sensor and the linear stage, which positioned the ISAC, were operated from a custom-developed graphical user interface written in Python using PyQt.

After each displacement of the reference magnet, the output (OP) of the control loop was updated in real time with OP_new_ = OP_previous_ + *K*_P_ × (SP − FB), with the feedback FB being the measured sensor signal, SP being the desired sensor signal (set point), and the proportional constant *K*_P_ = 10^8^ dp V^−1^, where dp refers to displacements units that are specific to the used linear motor.

### Generation of reference magnetic fields and collection of sensor data

Driving signals for electromagnetic coils used for the tracking of ISAC were generated as follows: Wave files (.WAV, 44.1-kHz sample frequency, 15-bit resolution) with two audio channels were generated to contain sinusoidal signals with a 90° phase offset. A commercial USB sound card (StarTech) generated the analog audio signal, which was applied to a commercial audio amplifier (E-400, t-amp). The amplifier output was tuned to generate an alternating current with an amplitude of 500 mA through the coils.

An instrument amplifier integrated circuit (INA121P, Texas Instruments) was used to amplify the differential output of the AMR sensor bridge with a gain of 100. Both the AMR sensor and the amplifier were supplied with 2.5 V from a source meter (Keithley 2614B). The steady-state current through the AMR sensor was tuned to 2 mA with an additional potentiometer connected in series. The amplified signal was recorded with an oscilloscope (PicoScope 5000, PicoTech) with a sampling rate of 1 to 10 MHz. The voltage drop over a 1-ohm resistor that was connected in series with one of the signal coils was measured and recorded using the same oscilloscope and used as reference.

### Processing of sensor data

Data acquisition and processing was carried out within a custom-written Python script with heavy usage of the NumPy and SciPy packages. Raw sensor and reference signals as recorded by the digital oscilloscope were band-pass–filtered to reduce lower- and higher-frequency distortions. Both signals were cross-correlated. The correlate peaked in the time bin that could be associated with the sensor signal phase shift, which was then converted into the position-dependent phase shift using the signal, an ADC frequency.

### Preparation of agar phantom

DI water in a beaker was placed on a hot plate and brought to a mild boil. Agar powder (1.5%_mass_) was added slowly under continuous stirring. The mixture was brought to a boil and removed from the hot plate. After cooling to about 60°C, the beaker with agar mixture was treated in an US bath to remove air bubbles. The still warm and liquid agar mixture was poured into the container where it was left to cure. Once cold, the agar block could easily slide out of the container, and the plastic tube was removed to leave a channel.

### Evaluation of US supported tracking experiment

#### Calibration

An ISAC was introduced stepwise (1 mm) into the channel over a total distance of 20 mm. At each position, the phase was determined five times (100-Hz reference signal, 300 periods) and the motor position was recorded. The data were then fitted with a function *d*Φ(*x*; *a*,*b*,*c*,*d*,*e*) = *a* × arctan(*b* × (*d* + (*x* − *c*))^3^/(*d* − (*x* − *c*))^3^) + *e*. The inverse relation *x*(Φ; *a*, *b*, *c*, *d*, *e*) was derived numerically and used to compute a position from a measured phase.

During the simultaneous US and magnetic tracking, an ISAC was introduced stepwise into the phantom channel (3- or 4-mm steps). At each step, a US image was obtained and the phase shift was determined. The reference motor position was corrected to remove a constant offset of 1.5 and 0.85 mm to compensate for minor displacements in between experiment runs.
