# Supplementary material for: Electronically integrated microcatheters based on self-assembling polymer films
Source: Sci Adv. 2021 Dec 17;7(51):eabl5408. doi: 10.1126/sciadv.abl5408 (PMC8682992; doi:10.1126/sciadv.abl5408)
Supplement: Supplementary file 1 — Supplementary Text Figs. S1 to S12 Legends for movies S1 to S6 [file sciadv.abl5408_sm.pdf]

Science Advances
AAAS

Supplementary Materials for

Electronically integrated microcatheters based on self-assembling polymer films

Boris Rivkin, Christian Becker, Balram Singh, Azaam Aziz, Farzin Akbar, Aleksandr Egunov, Dmitriy D. Karnaushenko, Ronald Naumann, Rudolf Schäfer, Mariana Medina-Sánchez*, Daniil Karnaushenko*, Oliver G. Schmidt*

*Corresponding author. Email: m.medina.sanchez@ifw-dresden.de (M.M.-S.); d.karnaushenko@ifw-dresden.de (D.K.); oliver.schmidt@main.tu-chemnitz.de (O.G.S.)

Published 17 December 2021, *Sci. Adv.* **7**, eabl5408 (2021)
DOI: 10.1126/sciadv.abl5408

The PDF file includes:

Supplementary Text
Figs. S1 to S12
Legends for movies S1 to S6

Other Supplementary Material for this manuscript includes the following:

Movies S1 to S6

Supplementary Text

Derivation of the relation between magnetic phase and position in 1D

The following model describes the position-phase relation for 1D displacement. The generalized discussion on 3D can be found below.
The reference magnetic fields emitted by electro-magnetic coils can be approximated as magnetic dipoles with the corresponding dipole field from a single source

$$\boldsymbol{B}(\boldsymbol{R}) = \frac{\mu_0}{4\pi r^2} \cdot \frac{3\boldsymbol{R}(\boldsymbol{M} \cdot \boldsymbol{R}) - \boldsymbol{M}r^2}{r^3} \qquad \text{SEq. 1}$$

where **B** is the magnetic field vector at position **R,** μ_0 the vacuum permeability 1.26 · 10^{-6} H m^{-1}, *r* the absolute distance $r = \sqrt{\mathbf{R}^2}$, and **M** is the dipole magnetization. Considering the filed along the axis through the coil center, the magnetic field is parallel to the axis and simplifies to

$$B(r) = \frac{\mu_0 \, M}{2\pi r^3} \qquad \text{SEq 2}$$

Magnetic phase encoded tracking requires two coils that face each other at a distance of 2·*d*. Assuming that a sensor at position *p* is separated from the sources by $r_1 = p + d$ and $r_2 = -(\mathrm{p} - \mathrm{d})$, the resulting field equals

$$B_{total}(p) = \frac{\mu_0}{2\pi}\left(\frac{M_1}{(p+d)^3} - \frac{M_2}{(p-d)^3}\right) \qquad \text{SEq 3}$$

Assuming that *p* = 0 is defined as the center point between the two coils, and M_1 and M_2 are the magnetizations of the two coils. The coils are supplied with alternating currents to generate an AC magnetization $M_1 = M_{1,0} \cdot \sin(\omega \cdot t)$ and $M_2 = M_{2,0} \cdot \sin(\omega \cdot t + 90°)$. With SEq. 3, this yields

$$B_{total}(p,t) = \frac{\mu_0}{2\pi}\left(\frac{M_{1,0}}{(p+d)^3} \cdot \sin(\omega \cdot \mathrm{t}) - \frac{M_{2,0}}{(p-d)^3} \cdot \sin(\omega \cdot \mathrm{t} + 90°)\right) \qquad \text{SEq. 4}$$

Using the harmonic addition theorem, which reads

$$a \cdot \sin(x + \alpha) + b \cdot \sin(x + \beta) = \sqrt{a^2 + b^2 + 2ab\,cos(\alpha - \beta)} \cdot \sin(x + \delta) \qquad \text{SEq. 5}$$

with

$$\delta = atan2(a \cdot \sin(\alpha) + b \cdot \sin(\beta), a \cdot \cos(\alpha) + b \cdot \cos(\beta)) \qquad \text{SEq. 6}$$

Applying the theorem to SEq. 4 results in

$$B_{total}(p,t) = \frac{\mu_0}{2\pi}\sqrt{\left(\frac{M_{1,0}}{(p+d)^3}\right)^2 + \left(\frac{M_{2,0}}{(p-d)^3}\right)^2} \cdot \sin(\omega t + \mathrm{d}\phi) \qquad \text{SEq. 7}$$

with the phase shift

$$\mathrm{d}\phi(p) = atan2\left(-\frac{M_{2,0}}{(p-d)^3}, \frac{M_{1,0}}{(p+d)^3}\right) = \arctan\left(-\frac{M_{2,0}}{M_{1,0}} \cdot \left(\frac{p+d}{p-d}\right)^3\right) \qquad \text{SEq. 8}$$

The last equality is true when both arguments within the atan2(x,y) are positive and can be ensured with the current direction or coil orientation. As denoted in SEq. 7, the resulting magnetic reference field is a sinus with a phase shift dϕ. The phase shift dϕ, which can be measured experimentally, is then directly linked to a position within the working distance according to SEq. 7 and 8. It should be noted that for $\frac{M_{1,0}}{M_{2,0}} = 1$, dϕ(*p*) has a steep gradient around *p* = 0. However, this gradient shifts for $\frac{M_{1,0}}{M_{2,0}} \neq 1$ towards the weaker signal source, indicating the possibility to

control the phase gradient, and thus generate regions with high spatial tracking resolution throughout the working volume at desired locations.

Derivation of the relation between magnetic phase and position in 3D

Magnetic phase-encoded tracking can be performed in 3D to navigate tools with 5 DOF.

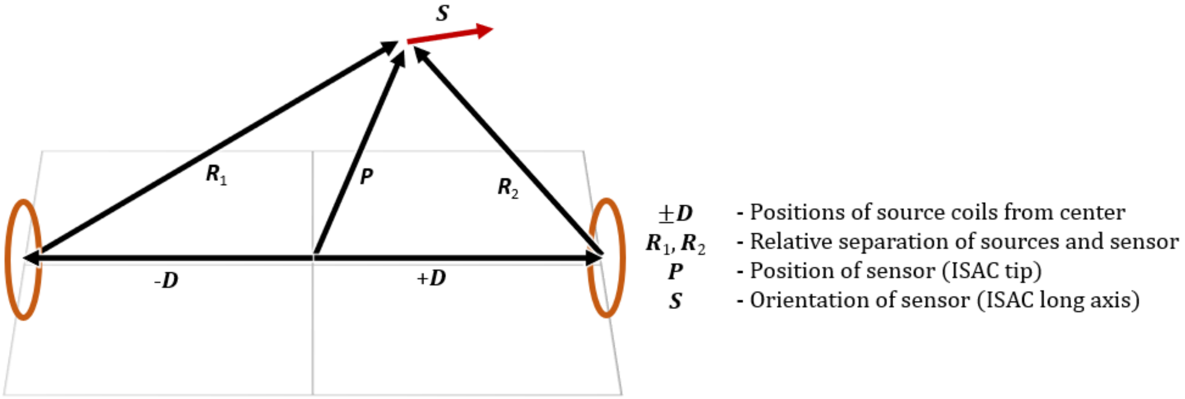

The total field is again a superposition of two reference fields $\boldsymbol{B} = \boldsymbol{B_1}+\boldsymbol{B_2}$, that are generated by two source coils at positions $\pm\boldsymbol{D}$. The relative separation between source and sensor are $\boldsymbol{R_1} = \mathbf{P} + \mathbf{D}$ and $\boldsymbol{R_2} = \boldsymbol{P} - \boldsymbol{D}$. Using the model for magnetic dipoles in 3D (SEq. 1), the constituent fields read

$$\boldsymbol{B}_{1/2}(\boldsymbol{P};\boldsymbol{D}) = \frac{\mu_0}{4\pi}\cdot\frac{3(\boldsymbol{P}\pm\boldsymbol{D})\left(\boldsymbol{M}_{1/2}\cdot(\boldsymbol{P}\pm\boldsymbol{D})\right)-\boldsymbol{M}_{1/2}\,(\boldsymbol{P}\pm\boldsymbol{D})^2}{(\boldsymbol{P}\pm\boldsymbol{D})^5} \qquad \text{SEq. 9}$$

where $\boldsymbol{M}_{1/2}$ are the magnetizations of the source coils. By design, the source magnetizations align with the source position vector $\boldsymbol{D}$. When supplied with two phase-shifted currents as above, the source magnetizations are

$$\boldsymbol{M}_1 = M_{1,0}\cdot\sin(\omega t)\frac{\boldsymbol{D}}{\sqrt{D^2}} \qquad \text{SEq. 10}$$

$$\boldsymbol{M}_2 = M_{2,0}\cdot\sin(\omega t+90°)\frac{\boldsymbol{D}}{\sqrt{D^2}} \qquad \text{SEq. 11}$$

where $M_{1,0}$ and $M_{2,0}$ indicate the magnetization amplitude that is defined by the supply current. When measuring a weak magnetic field (< 1mT), the integrated AMR sensor detects the projection onto the sensing axis, i.e. the component of the field vector that aligns with the sensitive axis. In ISACs, the AMR sensing axis aligns with long catheter axis, i.e. its orientation. The sensor output O is thus proportional to the dot-product of an external field $\boldsymbol{B}(\boldsymbol{P})$ at the sensor position $\boldsymbol{P}$ and the ISAC orientation $\boldsymbol{S}$

$$O = s\cdot\boldsymbol{B}(\boldsymbol{P})\cdot\boldsymbol{S} \qquad \text{SEq. 12}$$

with the constant s linking sensor properties and electronic output, and using the sensor orientation described by the unit vector $\boldsymbol{S}$. Inserting SEq 9 to 11, the sensor output is

$$O \;=\; s\cdot\boldsymbol{B}(\boldsymbol{P})\cdot\boldsymbol{S} = \; s\cdot(\boldsymbol{B_1}(\boldsymbol{P},t;\boldsymbol{D})\cdot\boldsymbol{S}) + (\boldsymbol{B_2}(\boldsymbol{P},t;\boldsymbol{D})\cdot\boldsymbol{S})$$

$$= \frac{s\,\mu_0 M_{1,0}\sin(\omega t)}{4\pi}\left(\frac{3(\boldsymbol{P}+\boldsymbol{D})\left(\frac{\boldsymbol{D}}{\sqrt{D^2}}\cdot(\boldsymbol{P}+\boldsymbol{D})\right)-\frac{\boldsymbol{D}}{\sqrt{D^2}}\,(\boldsymbol{P}+\boldsymbol{D})^2}{(\boldsymbol{P}+\boldsymbol{D})^5}\right)\cdot\boldsymbol{S}$$

$$+\frac{s\,\mu_0 M_{2,0}\sin(\omega t+90°)}{4\pi}\left(\frac{3(\boldsymbol{P}-\boldsymbol{D})\left(\frac{\boldsymbol{D}}{\sqrt{D^2}}\cdot(\boldsymbol{P}-\boldsymbol{D})\right)-\frac{\boldsymbol{D}}{\sqrt{D^2}}\,(\boldsymbol{P}-\boldsymbol{D})^2}{(\boldsymbol{P}-\boldsymbol{D})^5}\right)\cdot\boldsymbol{S} \quad \text{SEq. 13}$$

The relative orientation and position of the sensors and source coils can be summarized in the geometry factors g_1 and g_2 corresponding to source coil one and two, respectively

$$g_1 = \frac{s\cdot\mu_0}{4\pi}\sum\nolimits_{k\in(1,2,3)}\left(\frac{3(\boldsymbol{P}+\boldsymbol{D})_{\mathrm{k}}\left(\frac{\boldsymbol{D}}{\sqrt{D^2}}\cdot(\boldsymbol{P}+\boldsymbol{D})\right)-\frac{\boldsymbol{D}_{\mathrm{k}}}{\sqrt{D^2}}\,(\boldsymbol{P}+\boldsymbol{D})^2}{(\boldsymbol{P}+\boldsymbol{D})^5}\right)\cdot\boldsymbol{S}_{\mathrm{k}} \quad \text{SEq. 14}$$

$$g_2 = \frac{s\cdot\mu_0}{4\pi}\sum\nolimits_{k\in(1,2,3)}\left(\frac{3(\boldsymbol{P}-\boldsymbol{D})_{\mathrm{k}}\left(\frac{\boldsymbol{D}}{\sqrt{D^2}}\cdot(\boldsymbol{P}-\boldsymbol{D})\right)-\frac{\boldsymbol{D}_{\mathrm{k}}}{\sqrt{D^2}}\,(\boldsymbol{P}-\boldsymbol{D})^2}{(\boldsymbol{P}-\boldsymbol{D})^5}\right)\cdot\boldsymbol{S}_{\mathrm{k}} \quad \text{SEq. 15}$$

Summarizing SEq. 13 with the harmonic addition theorem SEq. 5 and 6, the expected sensor signal reads

$$O = \frac{s\cdot\mu_0}{4\pi}\sqrt{\left(\mathrm{M}_{1,0}\cdot g_1\right)^2+\left(\mathrm{M}_{2,0}\cdot g_2\right)^2}\cdot\sin(\omega t+\ \mathrm{d}\phi) \quad \text{SEq. 16}$$

with

$$\mathrm{d}\phi = \mathrm{d}\phi(\boldsymbol{P},\boldsymbol{S};\boldsymbol{D}) = \arctan\left(\frac{M_{2,0}}{M_{1,0}}\cdot\frac{g_2}{g_1}\right) \quad \text{SEq. 17}$$

Equation SEq. 17 implies that the phase shift $d\phi$, which can be measured experimentally, depends on the known positions of the source coils D = (D1, D2, D3), and the unknown position P = (P1, P2, P3) and orientation S = (S1, S2, S3) of the ISAC tip. This equation includes a total of six unknown variables, which is reduced by one, since by definition $S_3 = \sqrt{1-S_1^2-S_2^2}$. To identify the position and orientation of an ISAC with 5 DOF, i.e. solve SEq. 17 for P and S, at least five measurements of $d\phi$ with different source pairs, i.e. five different D, are required. In practice, additional source pairs should be used for redundancy. It should be noted that the special case D = (d, 0, 0), P = (p, 0, 0), and S = (1, 0, 0) where all vectors align with the x-axis and SEq. 17 reduces to the known 1D case found in SEq. 9.

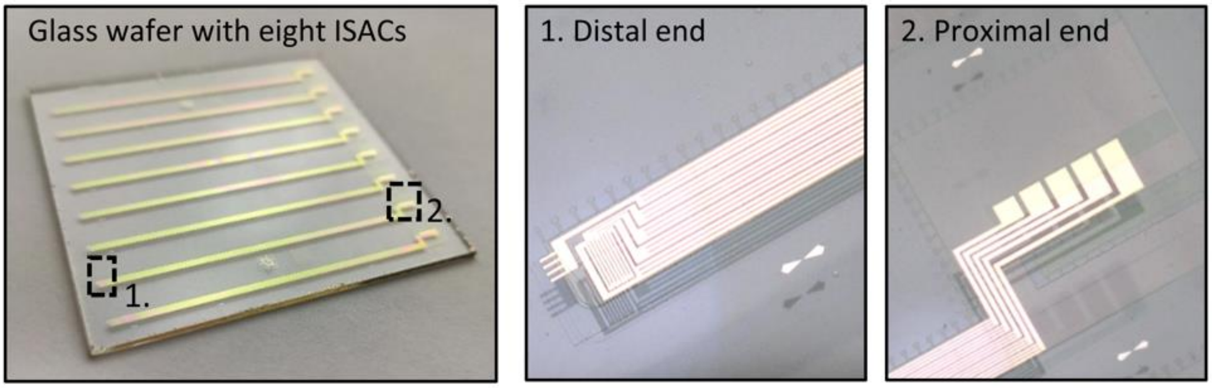

fig. S1. **Glass substrate carrying 8 ISACs.** The substrate size is 50 x 50 x 1 mm^3. Photo Credit: Boris Rivkin, Institute for Integrative Nanoscience, Leibniz IFW Dresden. Photo Credit: Boris Rivkin, Institute for Integrative Nanoscience, Leibniz IFW Dresden.

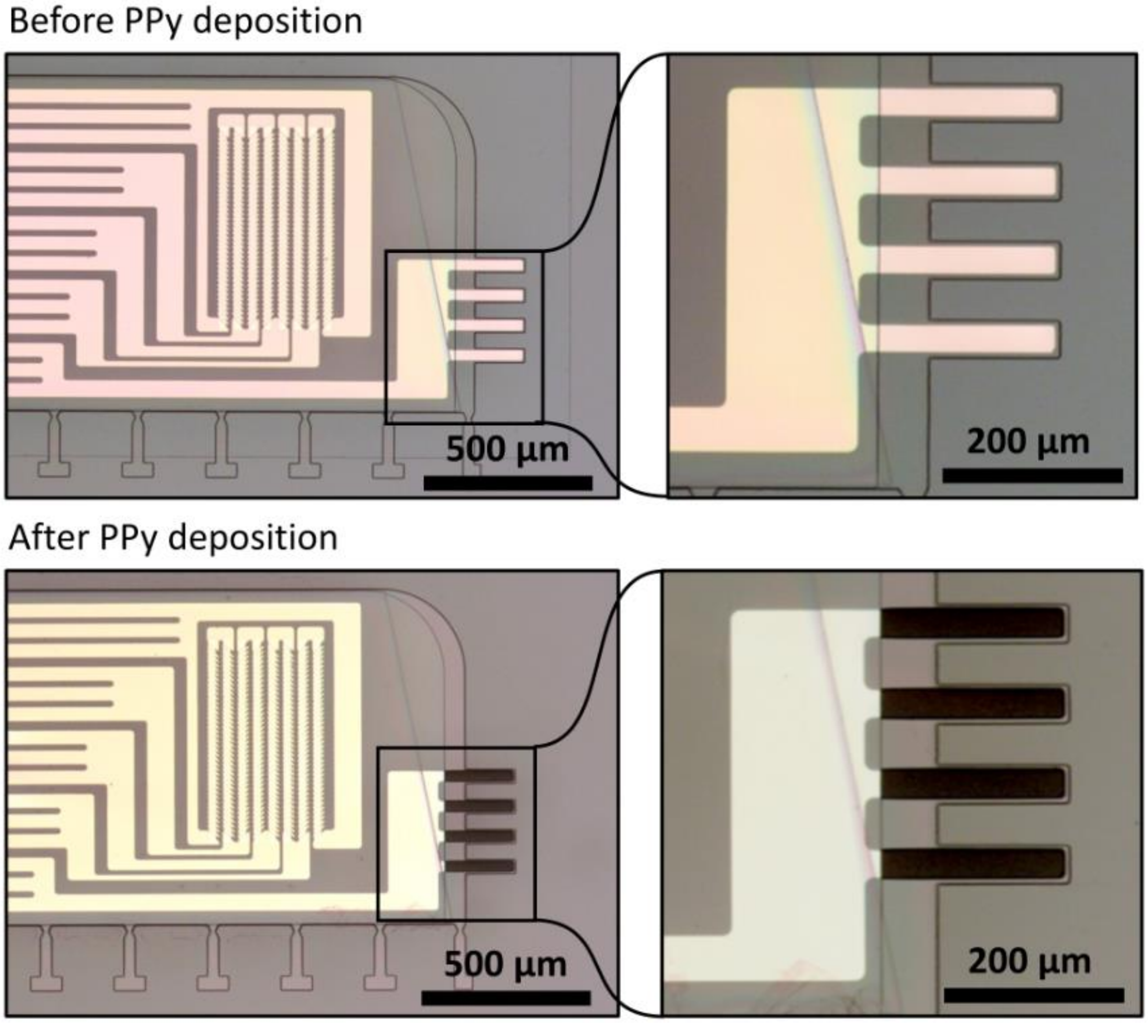

fig. S2. Micrograph of integrated manipulator digits. Four digits at the ISAC tip before (top) and after (bottom) electro-chemical deposition of Polypyrrole.

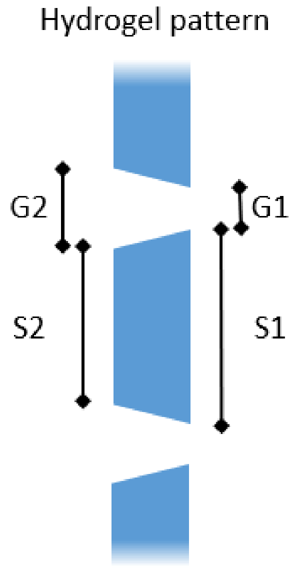

Type Nr.	S1 [mm]	S2 [mm]	G1 [mm]	G2 [mm]
1.	1	0.6	0.16	0.56
2.	2	1.6	0.16	0.56
3.	3	2.6	0.16	0.56
4.	4	3.6	0.16	0.56
5.	4.85	4.45	0.16	0.56

fig. S3. **Trapezoidal pattern of the hydrogel swelling layer measures.** ISACs with various HG patch sizes were realized as displayed in the table.

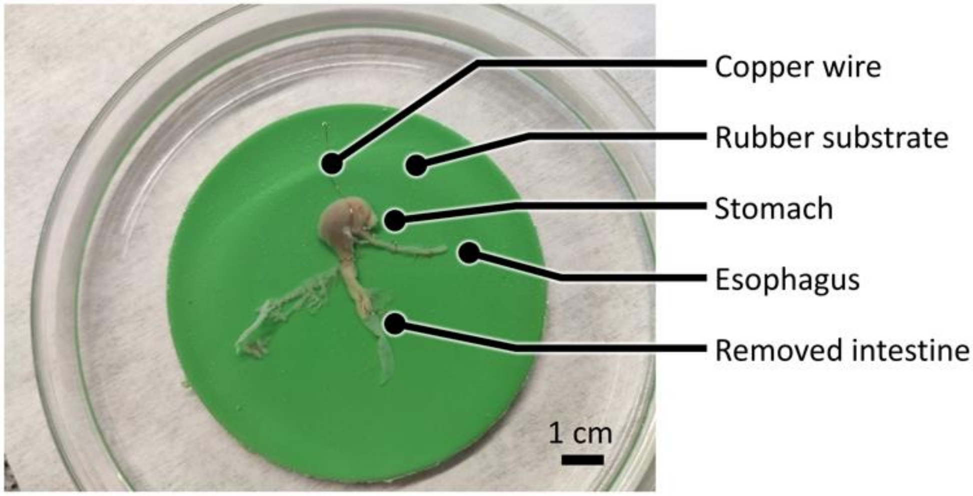

fig. S4. **Image of ex vivo mouse stomach.** The ex vivo mouse stomach with esophagus, mounted on green rubber in PBS solution. Photo Credit: Boris Rivkin, Institute for Integrative Nanoscience, Leibniz IFW Dresden.

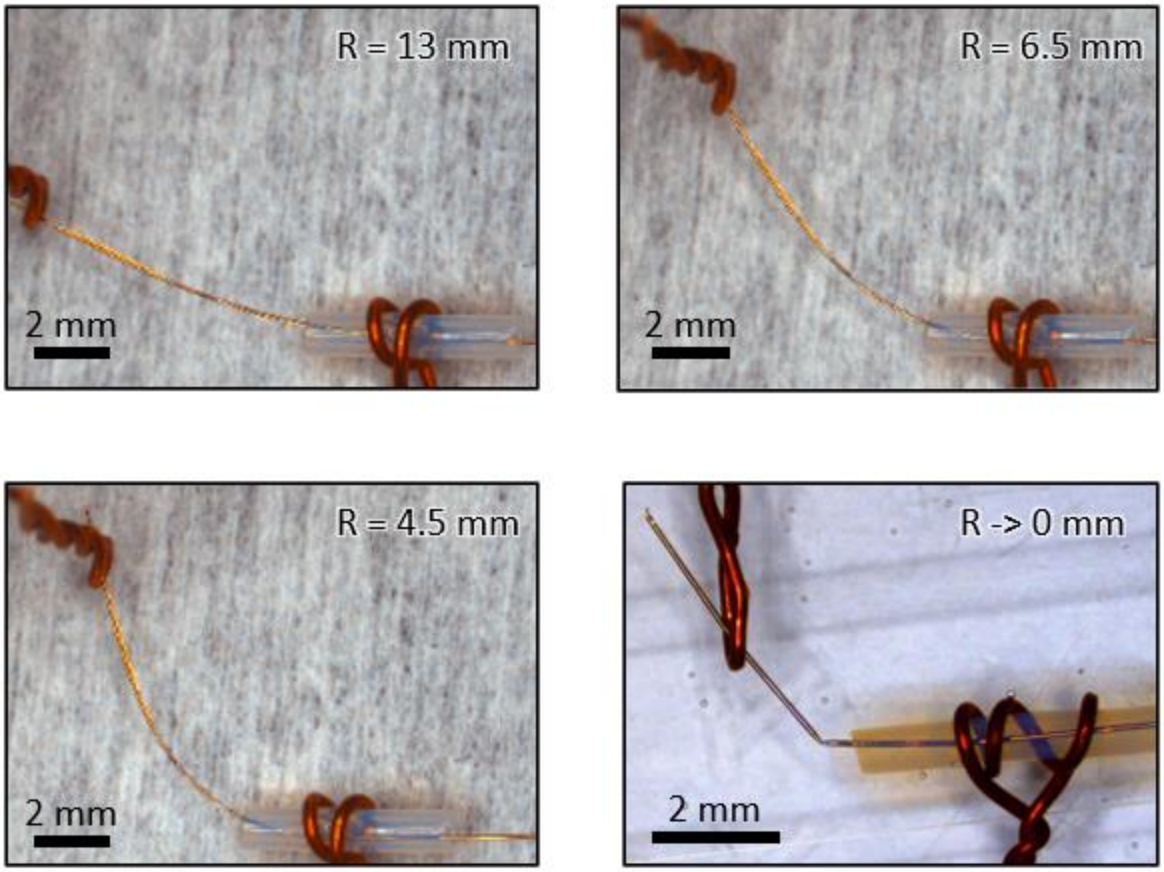

fig. S5. ISACs bent to various curvatures. Photo Credit: Boris Rivkin, Institute for Integrative Nanoscience, Leibniz IFW Dresden.

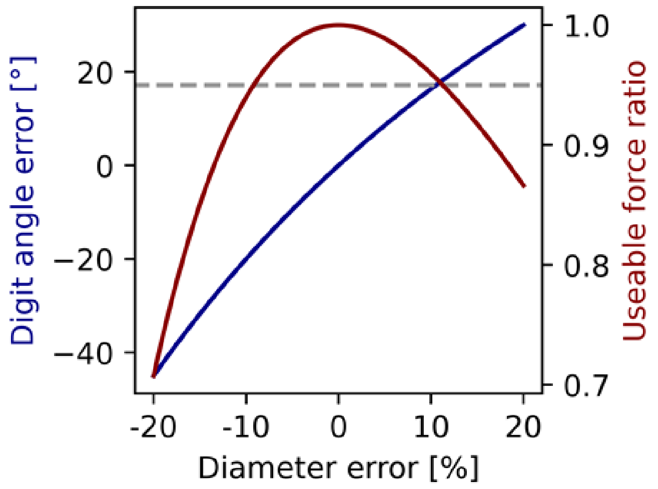

fig. S6. Estimated grasping force of misaligned manipulator digits.

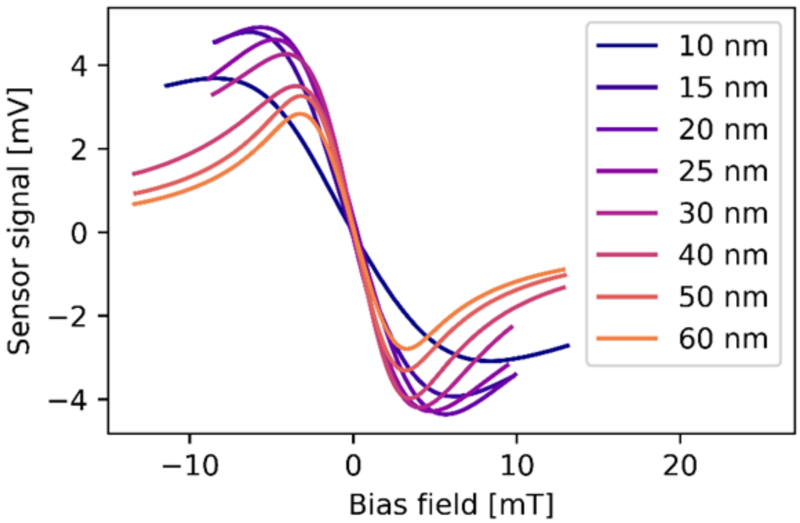

fig. S7. Response of AMR sensors with different NiFe layer thicknesses.

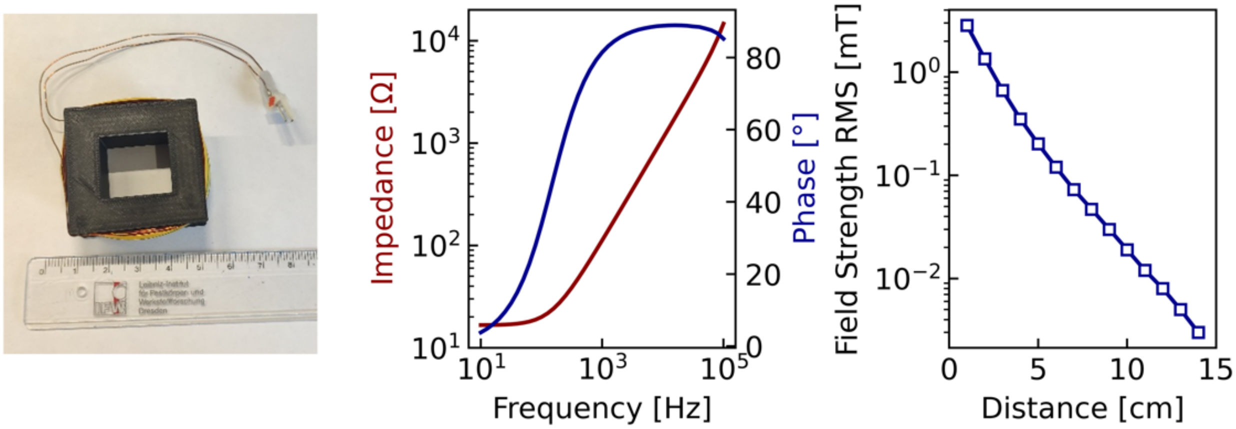

fig. S8. Characteristics of electro-magnetic coils use as reference for tracking. (**A**) Photograph of electro-magnetic coil. (**B**) Impedance and phase curve of a source coil. (**C**) RMS field strength generated by EM coil dependent on distance. Photo Credit: Boris Rivkin, Institute for Integrative Nanoscience, Leibniz IFW Dresden.

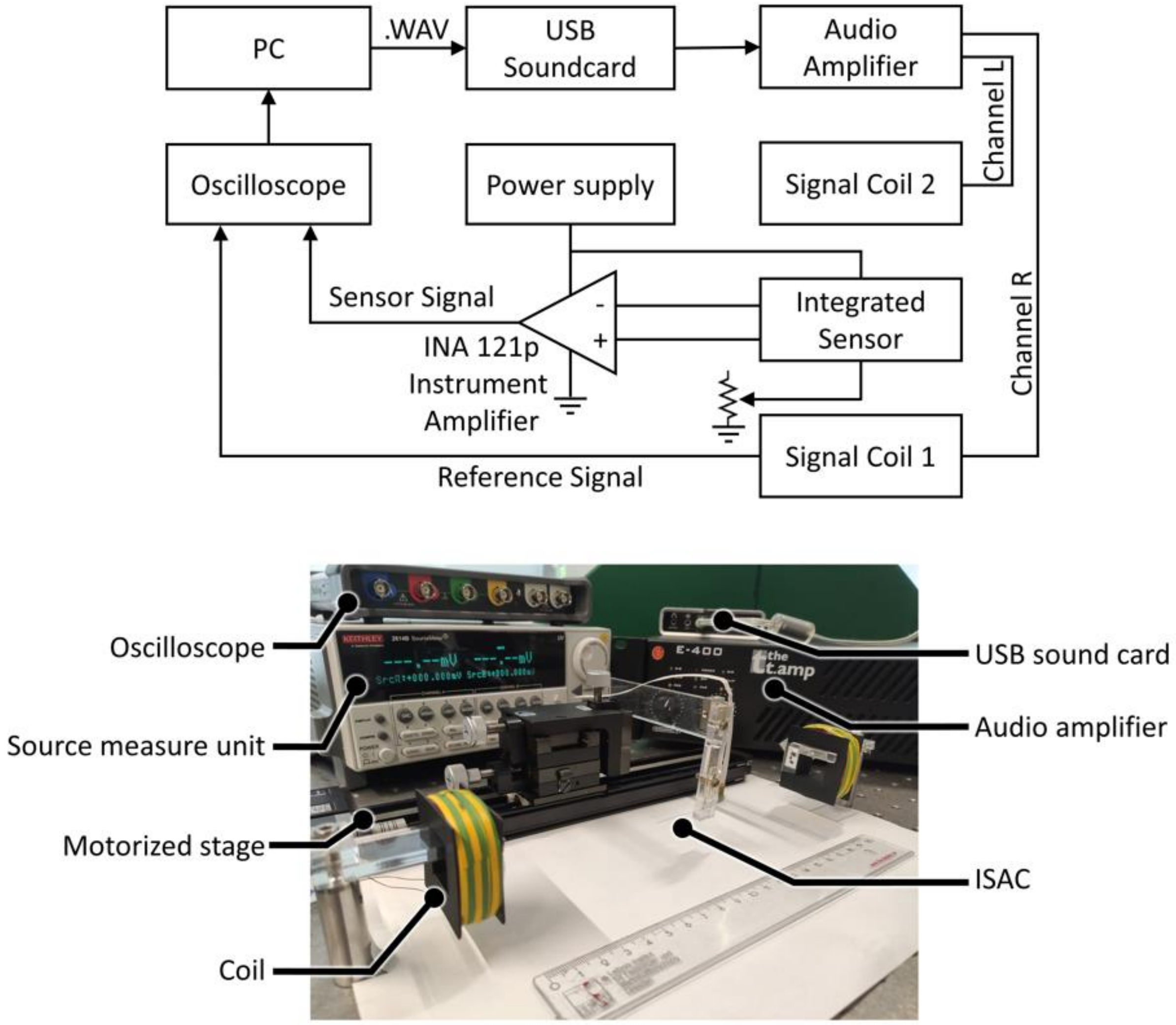

fig. S9. **Schematic and image of the experimental setup for magnetic phase encoded tracking.** Photo Credit: Boris Rivkin, Institute for Integrative Nanoscience, Leibniz IFW Dresden.

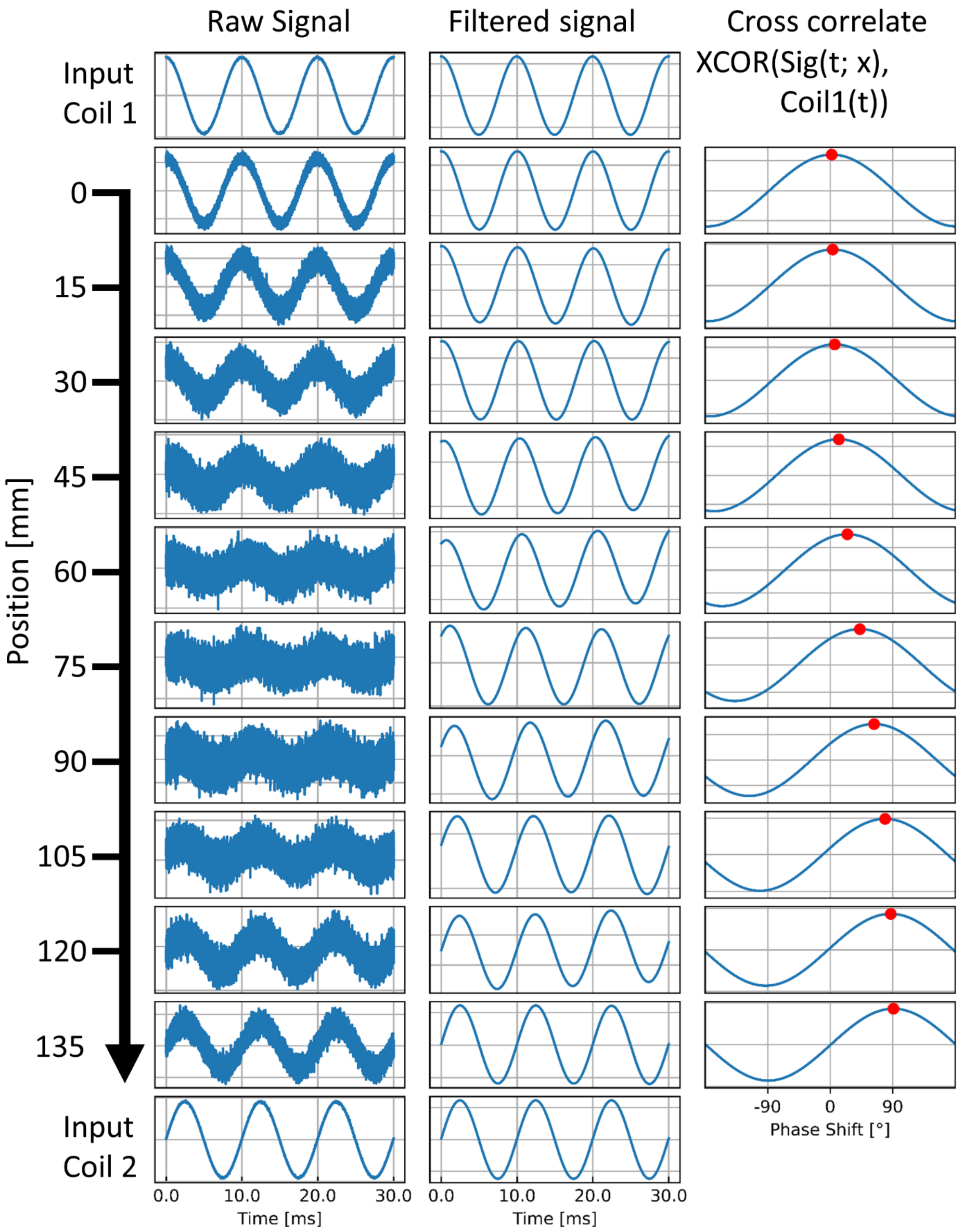

fig. S10: **Exemplary series of AMR sensor signal in raw and processed form.** AMR sensors signals as measured (left column) and filtered (center column) obtained at various locations along the center line. The inputs of the two reference field coils are displayed in the topmost and bottommost subplots in the left column. A phase shift between Input Coil 1 and the signals emerges from top to bottom. It is quantitatively assessed by cross-correlating Input Coil 1 and the filtered signal. The maximum (red dot) indicates the calculated phase shift.

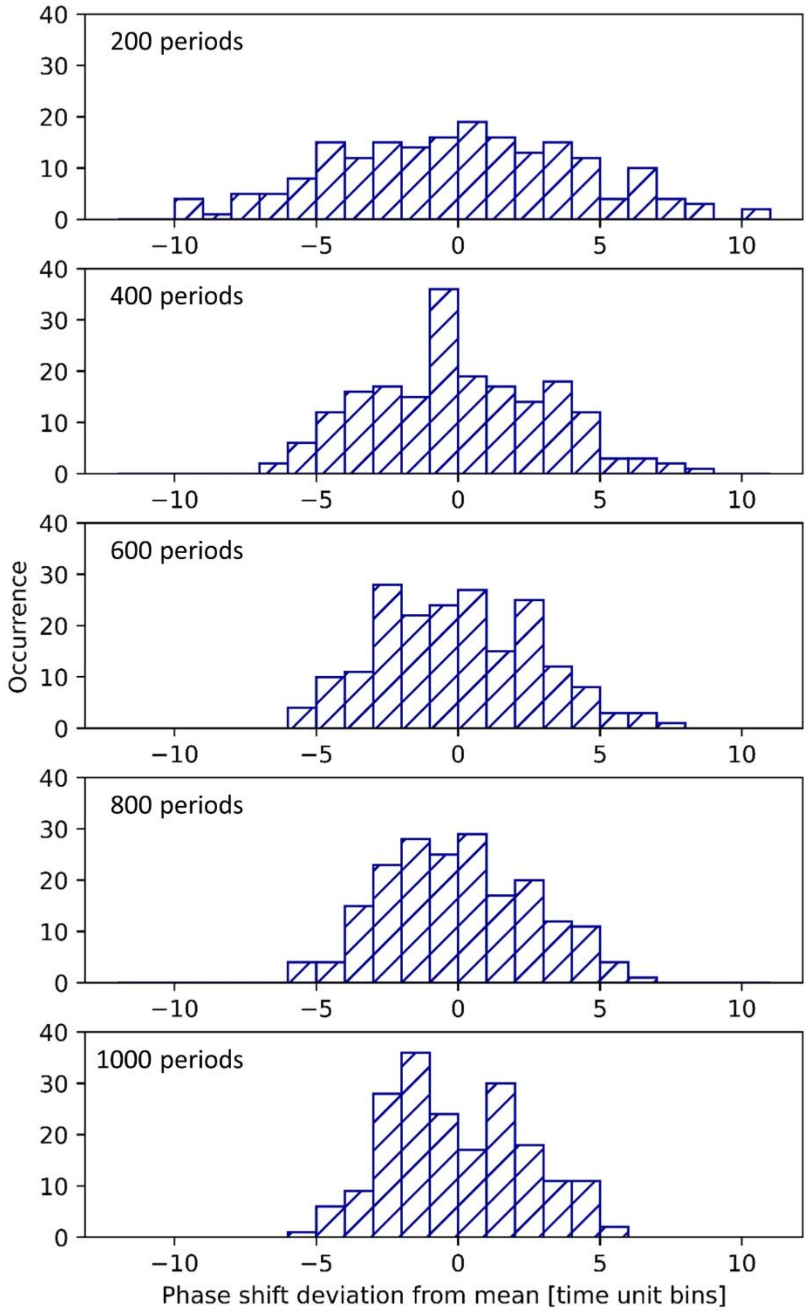

fig. S11: **Statistics of phase shifts measured with a static integrated AMR sensor.** Histograms of phase shifts measured with a static integrated AMR sensor for 200 times. Different portions of the signal were used to compute the phase shift via cross-correlation. Each time unit bin corresponds to 30 µm of position uncertainty.

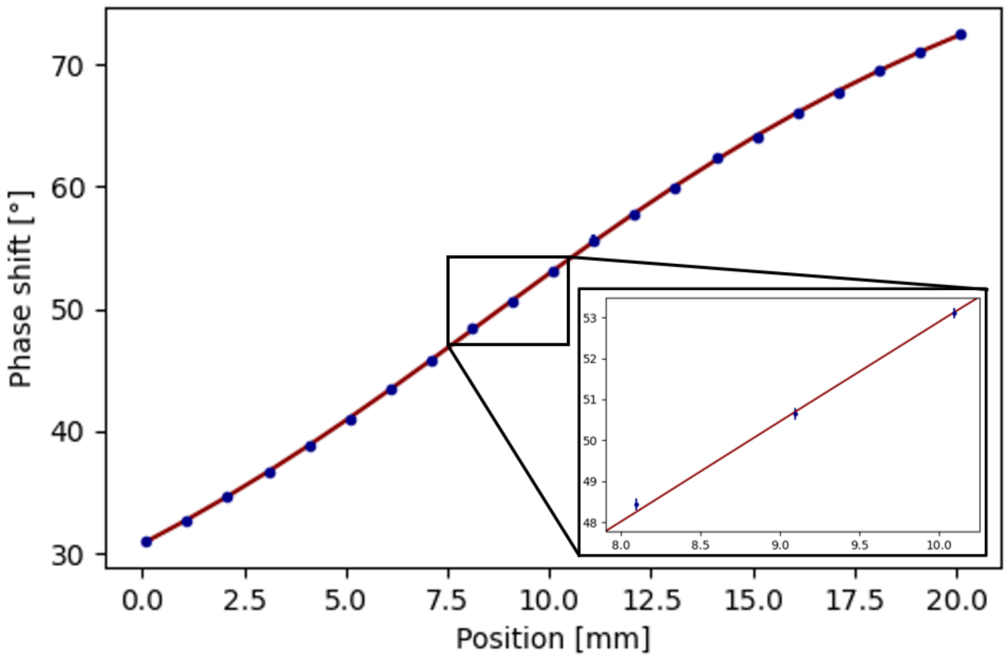

fig. S12: **Calibration curve and model fit for magnetic phase encoded tracking in agar phantom channel.** Data points are an average over 5 measurements, the inset allows to see the error bars.

Supplementary Movie S1: An ISAC is introduced through an artificial channel to deliver a liquid into a cavity.
Supplementary Movie S2: Insertion into curved channel and mechanical stress test.
Supplementary Movie S3: An ISAC is introduced through an ex vivo mouse esophagus into the stomach.
Supplementary Movie S4: A micro-particle is retrieved out of a model channel using the integrated manipulator if an ISAC.
Supplementary Movie S5: Feedback-driven position control of an ISAC with a reference magnet.
Supplementary Movie S6: US imaging of the insertion of an ISAC into the agar model channel.
